# The Effect of Foliar Application of Melatonin on Changes in Secondary Metabolite Contents in Two *Citrus* Species Under Drought Stress Conditions

**DOI:** 10.3389/fpls.2021.692735

**Published:** 2021-09-08

**Authors:** Marziyeh Jafari, Alireza Shahsavar

**Affiliations:** Department of Horticultural Science, College of Agriculture, Shiraz University, Shiraz, Iran

**Keywords:** drought stress, essential oil, flavonoid, lime species, melatonin, polyphenols

## Abstract

Plant secondary metabolites are compounds that play an important role in plant interactions and defense. Persian lime and Mexican lime as the two most important sour lime varieties with high levels of secondary metabolites, are widely cultivated in tropical and subtropical areas. Melatonin is a pleiotropic molecule that plays a key role in protecting plants against drought stress through regulating the secondary metabolite biosynthesis pathway. This study was performed as a factorial experiment consisting of three factors in a completely randomized design (CRD), including four concentrations of melatonin (0, 50, 100, and 150 μM), three levels of drought stress [100% (control), 75% (moderate stress), and 40% (severe stress) field capacity (FC)], and two *Citrus* cultivars. The experiment was conducted for 60 days in a greenhouse condition. Based on the results of this study under severe drought stress, melatonin-treated crops had higher total flavonoid and total phenolic contents than the untreated crops. The highest level of essential oils components was observed on 100 μM foliar application of melatonin under severe drought stress in both varieties. The main component of the essential oil was limonene in both *Citrus* species. Moreover, based on the analysis of the results, hesperidin was the main polyphenol in both varieties. Since the use of melatonin often increases the production of secondary metabolites, this study can be considered as a very effective method for controlling the adverse effects of drought stress in citrus for both industrial and horticultural aims.

## Introduction

Plant secondary metabolites are a wide range of biologically active substances that are remarkably important for plant growth and development (Ashraf et al., 2019). Although secondary metabolites are not essential for cells to live, these biomolecules play a pivotal role in cell and environment interactions (Hatcher et al., 2020). Indeed, secondary metabolite synthesis can be considered as functional and structural stabilization of plants to cope with stressful conditions during growth and development through a signaling pathway (Erb and Kliebenstein, 2020; Hatcher et al., 2020). Several studies in different plants such as hypericon (*Hypericum polyanthemum*) (de Matos Nunes et al., 2014), *Lamiaceae* plants (Kulak, 2020), black cumin (*Nigella sativa* L.) (Bayati et al., 2020), *Salvia* species (Bidabadi et al., 2020), and summer savory (*Satureja hortensis*) (Miranshahi and Sayyari, 2016) demonstrated that accumulation of secondary metabolites had been significantly increased under abiotic stresses, especially under drought stress. Alam et al. (2014) showed that essential oils, carotenoids, and polyphenols could be categorized as the most important secondary metabolites under drought stress in *Citrus* species. Flavonoids and non-flavonoids as the main classes of polyphenols play an important role in different physiological processes, such as preventing oxidative damage through synergism among phenolic compounds and their functions as radical scavengers (Lingua et al., 2016; Erb and Kliebenstein, 2020; Hatcher et al., 2020). Essential oils may consist of volatile terpenic biomolecules with the formula (C_5_H_8_)_n_ (*n* = 2, 3, and 4 demonstrating monoterpenes, sesquiterpenes, and diterpenes, respectively). The terpenoids can be considered oxygenated formatives of terpenes that include carbonyl and hydroxyl classes (Hesami et al., 2020a), and play a vital role in the fruit characteristic aroma (Hatcher et al., 2020) as well as possess anti-inflammatory, antioxidant, and anticancer activities (Bora et al., 2020).

Limes can be considered as the most important species from the genus *Citrus* (Rutaceae), which have a remarkable level of secondary metabolites. Persian lime (*Citrus latifolia* Tanaka) and Mexican or Key lime [*Citrus aurantifolia* (Christ.) Swingle] are widely cultivated for their antioxidant activity and high level of bioactive components such as anti-scurvy, appetite stimulant, antiseptic, digestive, anthelmintic, mosquito repellent, astringent, as well as treating fever, edema, cataract, stomach ailments, cold, headache, pharyngitis, earache, and pain (Apraj et al., 2011). Phenolic components as the biomolecules in citrus have a wide range of pharmaceutical activity (Wang et al., 2017). *Citrus* essential oils have been generally categorized as a complex mixture of about 400 components, including non-volatile and volatile compounds (Espina et al., 2011). Citral (geranial and neral), 1,8-cineole, limonene, β-bisabolene, α-terpineol, *p*-cymene, terpinen-4-ol, β-pinene, and 1,4-cineole can be considered as the most important essential oils of lime (Ranganna et al., 1983), which has broadly applied in different industries such as cosmetic, beverage, perfume, sweet, medicine, and chocolate (Bora et al., 2020).

Persian lime and Mexican lime as the two most important sour lime varieties are widely cultivated in tropical and subtropical areas, which are mostly faced with drought stress (Jafari and Shahsavar, 2020). Drought stress can be considered as one of the most serious stresses which result in changes in different processes such as growth parameters, transpiration, enzyme activity, photosynthesis, hormone metabolism, respiration, secondary metabolite production, as well as composition and yield of essential oils (Okunlola et al., 2017). Plants synthesize various secondary metabolites under unfavorable growth conditions that play an essential role in protecting plants from adverse effects of stresses. Some secondary metabolites as non-enzymatic antioxidant take part in defense responses against oxidative stress. Secondary metabolites such as essential oil, phenolic, and flavonoid compounds can scavenge free radicals by donating electron or hydrogen (Ashraf et al., 2019; Asghari et al., 2020).

Since drought stress in most cases results in a decrease in plant development, there is a dire need to find a solution to tackle this adverse impact (Yoosefzadeh Najafabadi et al., 2018; Hesami et al., 2020b; Jafari and Shahsavar, 2020). It is well documented that plant growth regulators play a pivotal role in regulating stress signaling and biochemical and physiological pathways (Wang et al., 2010; Hasanuzzaman et al., 2020). Exogenous application of phytohormones or plant bio-stimulators can be considered a powerful and useful approach to improve the protection and adaptability of crops against stressful environmental conditions (Ahmad et al., 2020). Melatonin is a molecule with multiple functions which has direct tasks in improving the performance of the mitochondrial electron transport chain, scavenging-free radicals, protecting antioxidant enzymes from oxidative damage, and increasing antioxidant enzyme activities (Reiter et al., 2010; Tan et al., 2012; Han et al., 2017; Ahmad et al., 2020). The positive effect of melatonin on ameliorating the adverse impact of abiotic stresses has been previously studied in different plants such as maize (*Zea mays*) (Huang et al., 2019), cucumber (*Cucumis sativus*) (Zhang et al., 2014), moldavian balm (*Dracocephalum moldavica* L.) (Kabiri et al., 2018), apple (*Malus domestica*) (Li et al., 2015), wheat (*Triticum aestivum*) (Ke et al., 2018), cotton (*Gossypium hirsutum* L.) (Hu et al., 2020), mutant barley (Li et al., 2016), maize (Sun et al., 2020), rapeseed cultivar (Khan et al., 2019), tomato cultivar “Qianxi” (Zhou et al., 2020) and soybean (*Glycine max* L.) (Wei et al., 2015). Recent studies reported that melatonin can promote secondary metabolite biosynthesis in plants under drought stresses. For example, Wei et al. (2019) found that exogenous melatonin modulated flavonoid contents in apple (*Malus hupehensis* L.) to respond salinity stress; Bistgani et al. (2019) found that foliar application of melatonin improved the total phenolic compounds in garden thyme (*Thymus daenensis* L.) leaves under salinity stress. By considering all the variations in secondary metabolites composition of citrus, it is necessary to have a comprehensive study on secondary metabolite production which will lead to an in-depth knowledge of their components. However, such detailed understanding is usually achieved through appropriate extraction methods and meticulously chromatographic analysis. Among various possible techniques, gas chromatography combined with mass spectrometry (GC–MS) can be considered a reliable method for analyzing citrus essential oil. Also, high-performance liquid chromatography (HPLC) can be used as a powerful method for analyzing citrus polyphenols (Jalali-Heravi and Parastar, 2011; Tranchida et al., 2012).

Although melatonin has been applied in agriculture to improve plant growth and development, the effect of exogenous application of melatonin on the production of secondary metabolites in important horticultural crops such as citrus has been rarely studied. Therefore, it is necessary to study the application of this promising molecule on secondary metabolite profiles. In the present study, it was hypothesized that drought would lead to secondary metabolite biosynthesis in two *Citrus* species, and exogenous melatonin application would increase secondary metabolite biosynthesis by regulating metabolic processes. Hence, the current research has been aimed to determine the effect of foliar application of melatonin on polyphenols and essential oils production of two *Citrus* cultivars (Mexican and Persian lime) under drought stress conditions.

## Materials and Methods

### Plant Material

The current study was performed at the research greenhouse of the College of Agriculture, Shiraz University, Shiraz, Iran, in September 2019. One-year-old seedlings of two *Citrus* cultivars, including Mexican lime (*C. aurantifolia* (Christ.) Swingle) and Persian lime (*C. latifolia* Tanaka) were transferred to plastic pots (5 kg, 33 cm diameter and 36 cm height) consisting of soil + leaf litter (3:2 w/w). The crops were kept in the greenhouse with 25 ± 2°C temperature and 80% relative humidity under natural photoperiod. The half-strength Hoagland nutrient solution three times a week was regularly applied to water the crops before the experiments.

### Experimental Design and Treatments

This study was performed as a factorial experiment consisting of three factors in a completely randomized design (CRD), including four concentrations of melatonin, three levels of drought stress, and two *Citrus* cultivars with four replications. Ethanol was used for dissolving melatonin (Sigma-Aldrich Chemie, Steinheim, Germany) and preparing different concentrations (0, 50, 100, and 150 μM). Also, Tween-20 (0.1%) as a surfactant was applied for the foliar application of melatonin. Different levels of drought stress including 100% field capacity (FC) (control), 75% FC (moderate stress), and 40% FC (severe stress) were considered as stress treatments. Drought stress treatment and melatonin foliar application were started simultaneously. A manual pump (30 mL per plant) was used for spraying melatonin solution three times per week for 60 days. The weight method was used for controlling the stress treatments. The collected data from the weight method was applied to identify the different amounts of water to use as a percentage of FC. To determine dry soil weight, 4 kg of soil were placed in the oven for 48 h at 103°C. The oven-dried soil was used for filling the pots. After that, the pots were completely watered to saturate the soils. The following equation was used to determine the percentage of FC:

(1) $$\mathrm{FC}\left(\%\right)=\left(\frac{\mathrm{Wet}\mathrm{soil}\mathrm{weight}\left(\mathrm{WSW}\right)-\mathrm{Dry}\mathrm{soil}\mathrm{weight}\left(\mathrm{DSW}\right)}{\text{DSW}}\right)100$$

The amount of water stored in the FC condition was calculated after deducting the weight of the dry soil and the pot. Accordingly, different drought stress levels (40, 75, and 100% FC) were determined (Pourmeidani et al., 2017).

### Methanolic Extract Preparation for Determining Total Flavonoid and Phenol Contents

To facilitate the extraction, 1 mL 70% (v/v) methanol was used to homogenize and pulverize the fresh leaves (1 g per treatment). To obtain the supernatant (methanol extract) for determining flavonoid and phenol contents, the extracts after 30 min of incubation on the ice were centrifuged for 10 min at 10,000 rpm under 22°C temperature.

### Total Phenolic Content

Gallic acid (Sigma-Aldrich Chemie, Steinheim, Germany) as a standard phenolic compound and Folin–Ciocalteu reagent were used to determine the total phenolic contents of both cultivars. For preparing the reaction mixture, 500 μL of 20% of NaHCO_3_, 50 μL of the extract solution, 100 μL of 10% Folin–Ciocalteu’s reagent dissolved in water, and 1 mL of distilled water were mixed. Also, a blank solution was prepared. The samples were inoculated in a thermostated bath at 45°C for 45 min, and the solution absorbance was obtained at 720 nm. The total phenolic content of the extracts was shown as milligrams of gallic acid equivalents per gram of extracts (mg GAEs/g^–1^ ex) by calibrating the curve with gallic acid. Four replications in each sample were used for analyzing samples (Velioglu et al., 1998).

### Total Flavonoids Content

The aluminum chloride colorimetric technique was used to determine the flavonoid contents. A standard solution (20 μL) of quercetin (1–200 μg mL^–1^) or a particular volume of extracts was diluted with 10 μL of 5% AlCl_3_ and 60 μL of methanol. After that, the mixture was dissolved in 10 μL of 0.5 M potassium acetate, and the total volume was made up to 200 μL by adding distilled water. The mixture solution was incubated at room temperature for 30 min, followed by the determination of absorbance at 415 nm versus the blank. To obtain the calibration line, the same procedure was also replicated for the standard solution. The concentration of flavonoids (mg mL^–1^) was measured by using this calibration line. The outcomes were shown as milligrams per gram of quercetin equivalents (mg QE/g^–1^) of extract (Bahadori et al., 2015).

### Evaluation and Identification of Polyphenols Components Content by Using HPLC

#### Extraction

First, 2 mL of solvent (15% acetic acid + 85% methanol) were added to 0.2 g of pulverized fresh leaves. Since polyphenolic components are sensitive to light, the microtubes were covered with foil and kept for 24 h in dark freezer. The samples were placed in an ultrasonic bath (Bandelin, Germany) for 15 min in the dark at low temperature to separate the phenolic components from the tissue completely. After that, the microtubes were taken out of ultrasonic and located in a refrigerated centrifuge for 20 min at 10,000 rpm under 0°C temperature. The supernatant phase was removed from the samples and transferred to a new microtube, and finally, *N*-hexane was added to the new microtube. The microtubes were vortexed for 10–15 s and centrifuged again for 10 min at 0°C at 10,000 rpm. A two-phase solution was produced, which was the polyphenolic sub-phase. The microtubes were placed on the refrigerator until inoculated into the HPLC (Justesen et al., 1998; Gholami et al., 2018). All the standards were bought from Sigma Chemical Company.

#### High Performance Liquid Chromatography Analysis

High performance liquid chromatography was used to recognize polyphenols content for all the samples. An Agilent Technologies 1200 series HPLC, Germany instrument equipped with a vacuum degasser, UV-Vis photodiode array (DAD) detector, autosampler, binary pump, and analytical column (Inertsil ODS-3 5 μm 4.6 × 150 mm; GL Sciences Inc., Tokyo, Japan) was used to load an aliquot of sample extract. A syringe filter (0.22 mm) was used for filtering the extracts and then, the samples were directly injected into a C18 guard column by using a 10 mL fixed loop. Phenolic components were completely separated on an XDB-C18 column (4.6 mm 250 mm, 5 mm, Kromasil, Sweden) kept at 30°C. When the mobile phase contained methanol (60%) and water (40% acidified with 1% formic acid), isocratic elution mode with a flow rate of 1 mL/min was applied. The oven temperature and the total running time were 30°C and 45 min, respectively.

**FIGURE 1:**
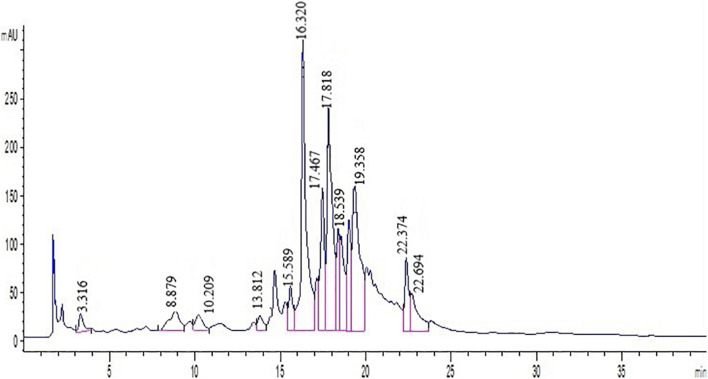
Chromatogram of standard mixture at 280 nm analyzed by HPLC. Peak numbersare: Quercetin (21.6), Vanillin (13.5), Ferulic acid (16.3), Hesperidin (18.5), Hesperetin (22.3), Catechin (8.8), Chlorogenic acid (10.2), Coumaric acid (15.6), Gallic acid (13.8), Nobiletin (11.7), Eriocitrin (22.6), Neohesperidin (17.8), Naringin (21.3), Rutin (3.31).

Polyphenols were identified by comparing the retention times of peaks in extracts to samples of standards at 280 nm (Figure 1). Phenolic components such as nobiletin, gallic acid, hesperetin, catechin, naringenin, *p*-coumaric acid, hesperidin, caffeic acid, naringin, rutin, epicatechin, ferulic acid, and quercetin were identified by employing several standard compounds.

### Essential Oil Isolation by the Hydro-Distillation Method

An all-glass Clevenger-type apparatus was employed to extract the essential oils (W/W%) based on the European Pharmacopoeia method (Formisano et al., 2015; Mechergui et al., 2016). The leaves (50 g) of both cultivars were washed under tap water to remove the surface contamination and then cut into small segments to increase their surface area. After that, the leaves were placed into a box containing 2000 mL distilled water. The hydrodistillation was performed for 3 h. The isolated essential oils volume was calculated. After that, anhydrous sodium sulfate was used for drying the essential oils, and then the samples were sealed and kept in the refrigerator in dark vials at 4°C. The following equation was used for calculating essential oil yield obtained from each cultivar:

(2) $$\text{O}\mathrm{il}\mathrm{yield}\left(\%\right)=\frac{\mathrm{Mass}\mathrm{of}\mathrm{essential}\mathrm{oil}\left(g\right)}{\mathrm{Mass}\left(g\right)\mathrm{of}\mathrm{plant}\mathrm{material}\left(\mathrm{dry}\mathrm{weight}\right)}\times 100$$

Gas chromatography and GC–MS were used to analyze the essential oil components (Duymuş et al., 2014).

### Procedure of Essential Oil Analysis

An Agilent gas chromatograph series 7890B armed with a flame ionization detector (FID) was used for the GC analysis. The analysis was performed on fused silica capillary HP-5 column (30 m × 0.32 mm i.d., with a film thickness of 0.25 μm). The detector and injector temperatures were kept at 280 and 250°C, respectively. *N*-hexane (ratio 1:100) was used for diluting the essential oil samples. Also, the samples were injected at a volume of 10 μL for each analysis. Nitrogen as the carrier gas was employed at a flow rate of 1 mL/min; oven temperature criterion was 65–215°C at the rate of 4°C/min, which was then processed to 245°C at the rate of 22°C/min, and ultimately, kept isothermally for 10 min.

A gas chromatograph (Agilent, 7955 A MSD) armed with a split/splitless injector, and an Agilent HP5-MS fused silica column (5% phenyl-methylpolysiloxane, 30 m × 0.25 mm i.d., film thickness 0.25 μm) was used for the GC–MS analysis. GC temperatures were set as previously mentioned. The extracts (10 μL) were injected into the column with a 1:50 split ratio. The quadrupole mass spectrometer was scanned over 40–550 amu with an ionizing voltage of 70 eV. Helium (99.999%) as the carrier gas was applied at a constant flow of 1.0 mL/min. Ion-source and injector temperatures were programmed at 280 nm and 240°C, respectively. The oven temperature criterion was 60–300°C at the rate of 3°C/min, which was then processed to 300°C at the rate of 22°C/min, and ultimately, kept isothermally for 10 min. The method of *n*-alkanes as standard was used for determining the retention indices for all compounds.

### Identification of Essential Oil Components

Essential oil compounds were identified using retention index, mass spectra, and compared with the proposed mass spectra by NIST libraries of GC connected to mass spectrometers and compared with standard compounds (Adams, 2007). The normalization approach of the GC/FID peak areas was used for calculating the percentage ratio of essential oil compounds.

### Statistical Analysis

Statistical analysis was performed for a factorial experiment with a CRD. Collected data were statistically analyzed using SAS software (SAS Institute, Cary, NC, United States), and Mean comparisons were applied using least significant difference (LSD) test at *P* ≤ 0.05. The results were expressed as mean ± standard deviation (SD). Mean values are presented of four biological measurements (*n* = 4) for total phenolic and flavonoid content, and for three biological measurements (*n* = 3) for essential oils and polyphenols analysis. To visualize the differences or similarities in the proportion of essential oil compounds under different treatments, Heml Heatmap Illustrator Software was used for generating the heatmaps.

## Results

### Evaluation of Total Phenol Content

**FIGURE 2:**
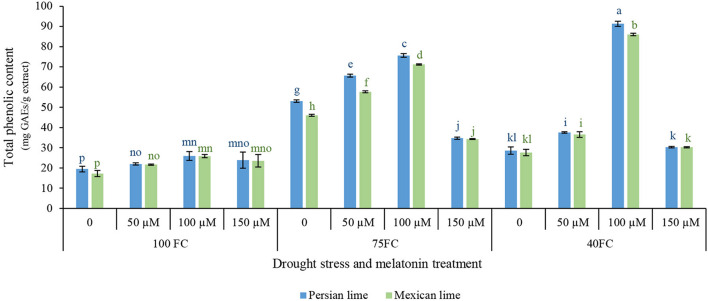
Effect of different concentrations of exogenous melatonin, *Citrus* species, and various levels of drought stress on total phenolic content. Means in each column followed by same letters at superscript are not significantly different according to LSD at *P* < 0.05. Values are given as mean ± SE (*n* = 4).

This study was aimed to determine the influence of drought stress and melatonin on the content of phenolic compound in Mexican and Persian lime. Results of statistical analysis indicated significant differences in the total phenolic content of the extracts of limes under different treatments (Figure 2). Results of this study showed that the extract from the two lime had higher total phenolic content under drought stress than unstressed condition. In addition, the results indicated that different levels of melatonin significantly affected the total phenolic content of the extracts (Figure 2). Generally, the results of this experiment showed that 100 μM melatonin under severe drought stress (40% FC) significantly enhanced the total phenolic content in both Persian lime (91.370 mg GAEs/g extract) and Mexican lime (86.08 mg GAEs/g extract) in comparison with the unstressed condition (25.826 mg GAEs/g extract for Persian lime and 17.217 mg GAEs/g extract for Mexican lime). Also, in moderate drought stress (75% FC), 50 and 100 μM melatonin showed a positive effect on the increase of total phenolic content compared to severe drought stress (40% FC). The lowest amount of total phenolic content (25.826 mg GAEs/g extract for Persian lime and 17.217 mg GAEs/g extract for Mexican lime) was observed in both species under unstressed condition without melatonin foliar application.

### Estimating Total Flavonoids Content

**FIGURE 3:**
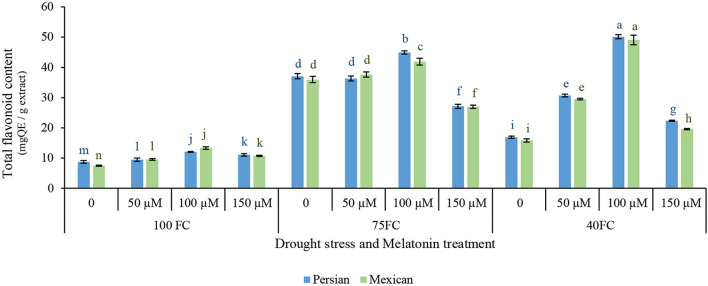
Effect of different concentrations of exogenous melatonin, *Citrus* species, and various levels of drought stress on total flavonoids content. Means in each column followed by same letters at superscript are not significantly different according to LSD at *P* < 0.05. Values are given as mean ± SE (*n* = 4).

The total flavonoid contents of the leaves in two cultivars were significantly affected by the interaction effects of drought stress and the exogenous application of melatonin (Figure 3). Total flavonoid content increased in two species under drought stress, but the rate of increase differed among the treatments. In two species, significant difference was observed between control and low stress, while from low to moderate stress, total flavonoid contents were dramatically increased. Severe drought stress increased total flavonoid contents compared to treatment under unstressed condition. Moderate stress further increased total flavonoid contents. Exogenous melatonin increased total flavonoid contents under drought stress, suggesting that it can increase the synthesis of secondary metabolites under drought stress. The application of 100 μM melatonin enhanced total flavonoid contents of Mexican lime (49.113 mg QE/g extract) and Persian lime (50.168 mg QE/g extract). The minimum total flavonoid contents (7.456 mg QE/g extract) were achieved from the unstressed Mexican lime without the application of melatonin (Figure 3).

### Polyphenols Identification and Quantification by HPLC

Direct HPLC injection and DAD detection at 280 nm was used to obtain polyphenol profiles in the leaf extract. Also, two peaks were identified at 325 nm in both cultivars. Since the results demonstrated that the peaks have a greater absorption at 280 nm, the chromatograms at 280 nm have been reported. The HPLC chromatograms achieved from standards and two cultivars extracts have been presented in Figure 1. As shown in Figure 1, Quercetin, Rutin, Vanillin, Naringin, Ferulic acid, Hesperidin, Neohesperidin, Eriocitrin, Hesperetin, Coumaric acid, Catechin, Nobiletin, and Gallic acid were detected by comparing their retention times with respective literature data and standards. Quantification of polyphenolic components from two cultivars (Mexican lime and Persian lime) has been obtained from a single relatively long run time (40 min). Rutin, in comparison with all peaks, had a better separation with a short time run (3.31).

The responses of major identified compounds to drought stress and foliar application of melatonin in two *Citrus* species were similar, where polyphenol components increased in two limes. The maximum percentage of the main polyphenol components was obtained in treatments under severe drought stress (40% FC) and 100 μM melatonin among polyphenols detected in Persian lime and Mexican lime. One hundred micromolars melatonin significantly increased the accumulation of polyphenol components. Although the interactions of melatonin and drought stress had significant effects on vanillin, hesperidin, gallic acid, nobiletin, eriocitrin, neohesperidin, and catechin in Mexican lime, there were no significant differences for the interactions of melatonin and drought stress on quercetin, ferulic acid, hesperetin, coumaric acid, chlorogenic acid, naringin, and rutin (Table 1). Moreover, the interactions of melatonin and drought stress had significant effects on quercetin, eriocitrin, hesperetin, coumaric acid, and nobiletin in Persian lime, while there were no significant differences for the interactions of melatonin and drought stress on vanillin, ferulic acid, catechin, chlorogenic acid, gallic acid, hesperidin, neohesperidin, naringin, and rutin (Table 2). Based on the results, hesperidin was the most abundant polyphenols in Mexican lime (14.52 ± 0.001 mg/g DW) (Table 1) and Persian lime (21.13 ± 0.003 mg/g DW) (Table 2) followed by eriocitrin (6.42 ± 0.01 mg/g DW in Mexican Lime and 13.36 ± 0.005 mg/g DW in Persian Lime (Tables 1, 2). Increasing the concentration of melatonin (150 μM) under stress and non-stress conditions had less effect on increasing polyphenol compounds than lower concentrations (50 and 100 μM melatonin). The lowest amount of these compounds was observed in the control treatment.

**TABLE 1:** Analysis of variance of polyphenols compound of Mexican lime under different concentrations of exogenous melatonin and various levels of drought stress.

Treatment	Quercetin	Vanillin	Ferulic acid	Hesperidin	Hesperetin	Coumaric acid	Chlorogenic acid	Gallic acid	Nobiletin	Eriocitrin	Neohesperidin	Catechin	Naringin	Rutin
	(mg/g DW)	(mg/g DW)	(mg/g DW)	(mg/g DW)	(mg/g DW)	(mg/g DW)	(mg/g DW)	(mg/g DW)	(mg/g DW)	(mg/g DW)	(mg/g DW)	(mg/g DW)	(mg/g DW)	(mg/g DW)
D0M0	0.99 ± 0.01^b^	0.122 ± 0.00^j^	0.50 ± 0.02^f^	9.31 ± 0.17^j^	0.919 ± 0.01^b^	0.273 ± 0.19^f^	0.341 ± 0.02^b^	0.261 ± 0.04^d^	0.562 ± 0.04^f^	4.17 ± 0.06^f^	0.710 ± 0.02^c^	0.813 ± 0.12^f^	3.11 ± 0.55^d^	2.14 ± 0.04^f^
D1M0	1.25 ± 0.05^b^	0.195 ± 0.008^e^	0.513 ± 0.004^d–f^	11.49 ± 0.05^g^	0.943 ± 0.01^a,b^	0.372 ± 0.01^a–e^	0.462 ± 0.01^a^	0.296 ± 0.009^b–d^	0.619 ± 0.001^c,d^	5.06 ± 0.03^c,d^	0.741 ± 0.04^b,c^	0.862 ± 0.02^b^	3.29 ± 0.05^c,d^	2.41 ± 0.06^d,e^
D2M0	1.22 ± 0.00^b^	0.197 ± 0.02^d,e^	0.515 ± 0.04^c–e^	11.40 ± 0.07^g^	0.948 ± 0.01^a,b^	0.365 ± 0.005^b–e^	0.449 ± 0.21^a,b^	0.301 ± 0.004^b,c^	0.616 ± 0.01^c–e^	5.12 ± 0.07^c,d^	0.738 ± 0.005^b,c^	0.851 ± 0.002^c^	3.34 ± 0.05^b–d^	2.42 ± 0.08^d,e^
D0M1	1.11 ± 0.01^b^	0.139 ± 0.02^i^	0.505 ± 0.07^e,f^	9.75 ± 0.003^i^	0.925 ± 0.07^b^	0.347 ± 0.00^e,f^	0.437 ± 0.02^a,b^	0.281 ± 0.16^c,d^	0.588 ± 0.12^d,e^	4.22 ± 0.01^e^	0.744 ± 0.03^b,c^	0.831 ± 0.01^e^	3.21 ± 0.06^c,d^	2.31 ± 0.02^d–f^
D1M1	1.71 ± 0.03^a^	0.203 ± 0.01^c,d^	0.523 ± 0.001^b–d^	13.20 ± 0.05^d^	0.958 ± 0.01^a,b^	0.387 ± 0.001^a–c^	0.464 ± 0.04^a^	0.312 ± 0.006^b,c^	0.621 ± 0.003^c,d^	5.94 ± 0.02^b^	0.761 ± 0.03^b,c^	0.863 ± 0.02^b^	3.76 ± 0.01^a,b^	2.72 ± 0.00^b,c^
D2M1	1.73 ± 0.00^a^	0.205 ± 0.02^c^	0.528 ± 0.03^b,c^	13.42 ± 0.08^c^	0.961 ± 0.01^a,b^	0.380 ± 0.01^a–d^	0.461 ± 0.01^a^	0.309 ± 0.007^b,c^	0.624 ± 0.01^c^	6.11 ± 0.02^a,b^	0.762 ± 0.05^b^	0.867 ± 0.005^b^	3.84 ± 0.00^a^	2.71 ± 0.01^b,c^
D0M2	1.12 ± 0.001^b^	0.153 ± 0.03^h^	0.507 ± 0.02^e,f^	10.81 ± 0.05^h^	0.952 ± 0.07^a,b^	0.354 ± 0.03^d–f^	0.437 ± 0.01^a,b^	0.287 ± 0.00^b–d^	0.596 ± 0.01^c–e^	4.94 ± 0.003^d^	0.749 ± 0.004^b,c^	0.854 ± 0.03^c^	3.22 ± 0.006^c,d^	2.35 ± 0.02^s^
D1M2	1.86 ± 0.01^a^	0.214 ± 0.004^b^	0.531 ± 0.001^b^	14.14 ± 0.007^b^	0.973 ± 0.004^a^	0.390 ± 0.03^a,b^	0.472 ± 0.02^a^	0.318 ± 0.003^b,c^	0.683 ± 0.004^b^	6.21 ± 0.006^a,b^	0.789 ± 0.004^b^	0.874 ± 0.01^a^	3.90 ± 0.001^a^	2.78 ± 0.03^a,b^
D2M2	1.94 ± 0.02^a^	0.222 ± 0.03^a^	0.544 ± 0.004^a^	14.53 ± 0.001^a^	0.975 ± 0.00^a^	0.398 ± 0.004^a^	0.487 ± 0.008^a^	0.321 ± 0.002^b^	0.688 ± 0.002^a^	6.42 ± 0.01^a^	0.793 ± 0.03^a^	0.877 ± 0.006^a^	3.97 ± 0.01^a^	2.89 ± 0.001^a^
D0M3	1.03 ± 0.007^b^	0.124 ± 0.006^j^	0.500 ± 0.009^f^	9.63 ± 0.01^i^	0.921 ± 0.03^b^	0.330 ± 0.002^g^	0.437 ± 0.08^a,b^	0.575 ± 0.004^a^	0.575 ± 0.007^e,f^	4.41 ± 0.00^e^	0.726 ± 0.003^b,c^	0.819 ± 0.001^f^	3.20 ± 0.006^d^	2.28 ± 0.01^e,f^
D1M3	1.67 ± 0.003^a^	0.160 ± 0.003^g^	0.521 ± 0.005^b–d^	12.30 ± 0.01^f^	0.936 ± 0.03^a,b^	0.360 ± 0.01^b–e^	0.444 ± 0.005^a,b^	0.301 ± 0.01^b,c^	0.632 ± 0.002^c^	5.24 ± 0.01^c,d^	0.754 ± 0.003^b,c^	0.841 ± 0.004^*d*^	3.56 ± 0.04^a–d^	2.55 ± 0.003^b–d^
D2M3	1.64 ± 0.002^a^	0.177 ± 0.001^f^	0.524 ± 0.003^b–d^	12.57 ± 0.03^e^	0.935 ± 0.01^a,b^	0.357 ± 0.003^c–f^	0.441 ± 0.006^a,b^	0.325 ± 0.025^b^	0.634 ± 0.04^b^	5.32 ± 0.004^c^	0.751 ± 0.03^b,c^	0.839 ± 0.01^d^	3.61 ± 0.01^a–c^	2.52 ± 0.005^c–e^
RT	21.6	13.5	16.3	18.5	25.4	15.6	10.5	13.3	11.7	24.6	3.86	8.3	21.3	3.31
*p*-Value														
Melatonin	0.0003	<0.0001	0.0003	<0.0001	0.0203	<0.0001	0.2324	<0.0001	<0.0001	<0.0001	<0.0001	<0.0001	0.0210	<0.0001
Drought	<0.0001	<0.0001	<0.0001	<0.0001	0.0277	<0.0001	0.1061	<0.0001	<0.0001	<0.0001	<0.0001	<0.0001	0.0002	<0.0001
Drought × melatonin	0.2889	<0.0001	0.2983	<0.0001	0.9912	0.0641	0.8563	<0.0001	<0.0001	<0.0001	<0.0001	<0.0001	0.0810	0.2368

*D0: irrigation at 100% Fc, D1: irrigation at 75% Fc, D2: irrigation at 40% Fc, M0 (0 μM melatonin), M1 (50 μM melatonin). M2 (100 μM melatonin) and M3 (150 μM melatonin). The data were sorted based on the retention time (RT) of components. Values are given as mean ± SD (n = 3). ^a−j^ Means in each column followed by the same letters at superscript are not significantly different according to LSD at P < 0.05.*

**TABLE 2:** Analysis of variance of polyphenols compound of Persian lime, under different concentrations of exogenous melatonin, and various levels of drought stress.

Treatment	Quercetin	Vanillin	Ferulic acid	Eriocitrin	Hesperetin	Catechin	Chlorogenic acid	Coumaric acid	Gallic acid	Nobiletin	Hesperidin	Neohesperidin	Naringin	Rutin
	(mg/g DW)	(mg/g DW)	(mg/g DW)	(mg/g DW)	(mg/g DW)	(mg/g DW)	(mg/g DW)	(mg/g DW)	(mg/g DW)	(mg/g DW)	(mg/g DW)	(mg/g DW)	(mg/g DW)	(mg/g DW)
D0M0	4.81 ± 0.003^b^	0.102 ± 0.002^a^	0.486 ± 0.02^c^	11.92 ± 0.03^h^	3.41 ± 0.003^e^	0.773 ± 0.01^e^	0.654 ± 0.01^a^	0.226 ± 0.02^e^	0.161 ± 0.005^c^	0.551 ± 0.009^g^	20.27 ± 0.003^d^	0.910 ± 0.005^i^	1.69 ± 0.006^g^	2.19 ± 0.01^d^
D1M0	5.36 ± 0.001^a^	0.104 ± 0.01^a^	0.512 ± 0.05^b,c^	12.37 ± 0.04^e^	3.90 ± 0.06^c,d^	0.816 ± 0.02^c–e^	0.651 ± 0.002^a^	0.322 ± 0.003^c^	0.196 ± 0.01^a,b^	0.627 ± 0.00^c^	20.76 ± 0.03^b,c^	1.24 ± 0.01^f,g^	2.21 ± 0.01^d–f^	2.45 ± 0.01^c,d^
D2M0	5.34 ± 0.003^a^	0.119 ± 0.002^a^	0.519 ± 0.01^b,c^	12.41 ± 0.02^d,e^	3.94 ± 0.01^c^	0.822 ± 0.01^b–e^	0.645 ± 0.004^a^	0.321 ± 0.005^c^	0.201 ± 0.003^a,b^	0.623 ± 0.003^c^	20.73 ± 0.01^b,c^	1.33 ± 0.01^e,f^	2.30 ± 0.04^c–e^	2.44 ± 0.01^c,d^
D0M1	4.92 ± 0.007^a^	0.106 ± 0.008^a^	0.505 ± 0.01^b,c^	12.14 ± 0.002^f,g^	3.65 ± 0.002^d,e^	0.796 ± 0.003^c–e^	0.632 ± 0.004^a^	0.241 ± 0.003^e^	0.181 ± 0.001^b,c^	0.571 ± 0.00^e^	20.43 ± 0.01^d^	1.14 ± 0.01^g,h^	2.10 ± 0.04^e,f^	2.32 ± 0.05^c,d^
D1M1	5.70 ± 0.002^a^	0.157 ± 0.004^a^	0.560 ± 0.001^a^	12.67 ± 0.006^c^	4.21 ± 0.006^a^	0.842 ± 0.01^a–d^	0.660 ± 0.01^a^	0.364 ± 0.007^b^	0.212 ± 0.01^a^	0.629 ± 0.001^c^	21.04 ± 0.003^a^	1.53 ± 0.002^c,d^	2.51 ± 0.001^a–d^	2.74 ± 0.00^a,b^
D2M1	5.74 ± 0.003^a^	0.163 ± 0.005^a^	0.562 ± 0.006^a^	12.71 ± 0.001^c^	4.23 ± 0.01^b^	0.853 ± 0.02^a–c^	0.641 ± 0.003^a^	0.372 ± 0.004^a,b^	0.209 ± 0.006^a^	0.626 ± 0.006^c^	21.09 ± 0.001^a^	1.65 ± 0.00^b,c^	2.65 ± 0.01^a–c^	2.76 ± 0.01^a,b^
D0M2	5.10 ± 0.003^a^	0.108 ± 0.005^a^	0.516 ± 0.001^b,c^	12.22 ± 0.01^f^	3.85 ± 0.02^c,d^	0.820 ± 0.003^b–e^	0.635 ± 0.002^a^	0.267 ± 0.004^d^	0.217 ± 0.01^a^	0.586 ± 0.01^d^	20.61 ± 0.001^c^	1.29 ± 0.005^e,f^	2.13 ± 0.006^e,f^	2.31 ± 0.003^c,d^
D1M2	5.82 ± 0.002^a^	0.182 ± 0.02^a^	0.570 ± 0.003^a^	13.23 ± 0.007^b^	4.71 ± 0.03^a^	0.876 ± 0.003^a,b^	0.670 ± 0.002^a^	0.385 ± 0.01^a^	0.218 ± 0.04^a^	0.681 ± 0.001^a^	21.19 ± 0.003^a^	1.72 ± 0.004^a,b^	2.73 ± 0.005^b^	2.88 ± 0.003^a^
D2M2	6.12 ± 0.001^a^	0.186 ± 0.01^a^	0.583 ± 0.01^a^	13.36 ± 0.005^a^	4.76 ± 0.01^a^	0.889 ± 0.01^a^	0.681 ± 0.003^a^	0.391 ± 0.002^a^	0.221 ± 0.004^a^	0.682 ± 0.005^a^	21.13 ± 0.003^a^	1.81 ± 0.003^a^	2.81 ± 0.002^a^	2.80 ± 0.001^a,b^
D0M3	4.87 ± 0.004^a^	0.104 ± 0.004^a^	0.503 ± 0.02^b,c^	12.06 ± 0.004^g^	3.52 ± 0.02^e^	0.789 ± 0.01^d,e^	0.632 ± 0.02^a^	0.241 ± 0.003^e^	0.213 ± 0.004^a^	0.563 ± 0.002^f^	20.40 ± 0.003^d^	1.06 ± 0.002^h^	1.94 ± 0.01^f,g^	2.28 ± 0.02^c,d^
D1M3	5.23 ± 0.005^a^	0.131 ± 0.001^a^	0.522 ± 0.005^b^	12.43 ± 0.003^d,e^	4.17 ± 0.02^b^	0.832 ± 0.002^b–e^	0.646 ± 0.003^a^	0.312 ± 0.01^c^	0.201 ± 0.02^a,b^	0.642 ± 0.00^b^	20.84 ± 0.01^b^	1.41 ± 0.002^d,e^	2.53 ± 0.00^a–d^	2.57 ± 0.001^b,c^
D2M3	5.21 ± 0.002^a^	0.124 ± 0.003^a^	0.526 ± 0.01^b^	12.46 ± 0.02^d^	4.13 ± 0.01^b^	0.830 ± 0.002^b–e^	0.649 ± 0.005^a^	0.314 ± 0.007^c^	0.206 ± 0.003^a,b^	0.644 ± 0.005^b^	20.82 ± 0.004^b^	1.35 ± 0.02^e,f^	2.41 ± 0.03^b–e^	2.53 ± 0.003^b,c^
RT	21.6	13.5	16.3	24.6	25.4	8.3	10.5	15.6	13.3	11.7	18.5	3.86	21.3	3.31
*p*-Value														
Melatonin	0.0020	0.6038	<0.0001	<0.0001	<0.0001	0.0035	0.9176	<0.0001	0.0006	<0.0001	<0.0001	<0.0001	0.0001	0.0016
Drought	0.0356	0.7457	<0.0001	<0.0001	<0.0001	0.0004	0.7531	<0.0001	0.0208	<0.0001	<0.0001	<0.0001	<0.0001	<0.0001
Drought × melatonin	0.0062	0.4341	0.2963	<0.0001	0.0453	0.9929	0.9935	0.0029	0.0509	<0.0001	0.4723	0.1993	0.8767	0.5782

*D0: irrigation at 100% Fc, D1: irrigation at 75% Fc, D2: irrigation at 40% Fc, M0 (0 μM melatonin), M1 (50 μM melatonin). M2 (100 μM melatonin) and M3 (150 μM melatonin). The data were sorted based on the Retention Time (RT) of components. Values are given as mean ± SD (n = 3). ^a−i^ Means in each column followed by the same letters at superscript are not significantly different according to LSD at P < 0.05.*

### Essential Oil Content

**FIGURE 4:**
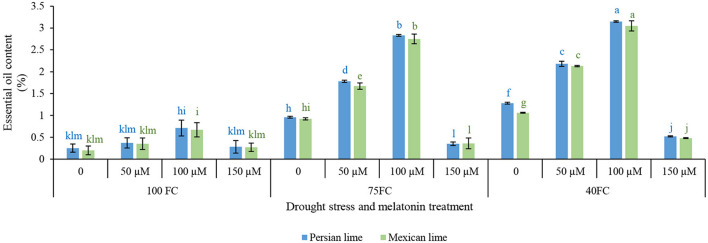
Effect of different concentrations of exogenous melatonin, *Citrus* species, and various levels of drought stress on essential oil content. Means in each column followed by same letters at superscript are not significantly different according to LSD at *P* < 0.05. Values are given as mean ± SE (*n* = 4).

The results showed that there was a significant difference at the 1% probability level for the interaction between examined factors affecting essential oil content (Figure 4). The means comparison (Figure 4) revealed that drought significantly increased essential oil content compared with well-watered control plants. Melatonin spraying under either stress or unstressed condition significantly increased essential oil content. The highest essential oil content (3.15% in Persian lime and 3.05% in Mexican lime) was obtained from the application of 100 μM melatonin. The application of melatonin at both concentrations (50 and 100 μM) significantly increased essential oil content compared with untreated plants. In general, melatonin at 100 μM in severe and moderate drought stress was more effective than 50 μM melatonin in improving essential oil content (Figure 4).

### Essential Oil Compositions

The composition of essential oil and their retention times produced in Persian lime and Mexican lime under different levels of drought stress and exogenous application of melatonin have been presented in Tables 3, 4. Through GC–MS and GC analysis, 61 essential oil components (99.5% of the total components) and 60 essential oil components (99.2% of the total components) were detected from leaves of Mexican lime and Persian lime, respectively (Tables 3, 4). There were some differences in the essential oil components between the two cultivars (Tables 3, 4).

**TABLE 3:** Essential oil compound of Mexican lime, different concentrations of exogenous melatonin, and various levels of drought stress.

Compounds	RT	D0M0	D1M0	D2M0	D0M1	D1M1	D2M1	D0M2	D1M2	D2M2	D0M3	D1M3	D2M3
α-Thujene	5.027	1.01 ± 0.003	1.23 ± 0.002	1.20 ± 0.003	1.1 ± 0.02	1.32 ± 0.003	1.25 ± 0.005	1.12 ± 0.004	1.44 ± 0.002	1.73 ± 0.003	1.07 ± 0.02	1.17 ± 0.003	1.14 ± 0.003
α-Pinene	5.627	3.12 ± 0.004	3.45 ± 0.02	3.37 ± 0.004	3.19 ± 0.02	3.53 ± 0.002	3.5 ± 0.01	3.23 ± 0.004	3.64 ± 0.05	3.72 ± 0.004	3.14 ± 0.01	3.30 ± 0.01	3.27 ± 0.01
Camphene	5.862	0.14 ± 0.01	0.53 ± 0.01	0.42 ± 0.002	0.25 ± 0.02	0.63 ± 0.01	0.58 ± 0.01	0.3 ± 0.004	0.71 ± 0.04	0.84 ± 0.002	0.20 ± 0.003	0.37 ± 0.02	0.34 ± 0.03
Sabinene	5.982	2.12 ± 0.02	2.49 ± 0.02	2.40 ± 0.002	2.18 ± 0.1	2.60 ± 0.01	2.54 ± 0.002	2.28 ± 0.003	2.76 ± 0.04	2.92 ± 0.001	2.13 ± 0.004	2.35 ± 0.003	2.32 ± 0.04
β-Pinene	6.188	12.37 ± 0.03	12.76 ± 0.006	12.6 ± 0.01	12.5 ± 0.01	12.90 ± 0.02	12.81 ± 0.005	12.55 ± 0.002	13.10 ± 0.00	13.2 ± 0.01	12.42 ± 0.01	12.6 ± 0.01	12.59 ± 0.002
β-Myrcene	6.486	1.92 ± 0.004	2.55 ± 0.002	2.42 ± 0.003	2.21 ± 0.1	2.66 ± 0.01	2.60 ± 0.004	2.3 ± 0.004	2.72 ± 0.01	2.81 ± 0.006	2.14 ± 0.01	2.37 ± 0.01	2.34 ± 0.01
α-Phellandrene	6.577	0.13 ± 0.01	0.62 ± 0.01	0.51 ± 0.002	0.27 ± 0.2	0.73 ± 0.004	0.67 ± 0.003	0.35 ± 0.003	0.84 ± 0.02	0.97 ± 0.01	0.19 ± 0.05	0.42 ± 0.001	0.39 ± 0.00
α-Terpinene	6.743	0.37 ± 0.00	0.56 ± 0.005	0.49 ± 0.01	0.37 ± 0.003	0.64 ± 0.003	0.61 ± 0.01	0.37 ± 0.002	0.72 ± 0.001	0.87 ± 0.002	0.37 ± 0.04	0.44 ± 0.001	0.41 ± 0.04
*o*-Cymene	7.395	1.30 ± 0.003	1.75 ± 0.001	1.67 ± 0.01	1.47 ± 0.02	1.87 ± 0.01	1.80 ± 0.02	1.53 ± 0.04	1.90 ± 0.005	1.99 ± 0.001	1.40 ± 0.001	1.60 ± 0.004	1.57 ± 0.003
Limonene	7.544	39.35 ± 0.004	58 ± 0.01	57.01 ± 0.004	45.3 ± 0.003	59.12 ± 0.02	58.39 ± 0.04	50.3 ± 0.02	60.5 ± 0.003	63.3 ± 0.005	40.3 ± 0.01	56.6 ± 0.00	50.41 ± 0.004
β-Ocimene	8.219	0.22 ± 0.002	0.49 ± 0.03	0.40 ± 0.005	0.28 ±	0.61 ± 0.03	0.54 ± 0.06	0.3 ± 0.01	0.73 ± 0.006	0.88 ± 0.001	0.25 ± 0.003	0.37 ± 0.002	0.34 ± 0.01
γ-Terpinene	8.34	15.44 ± 0.004	20.76 ± 0.002	20.48 ± 0.004	18.25 ± 0.01	20.94 ± 0.03	20.81 ± 0.006	19.94 ± 0.003	21.2 ± 0.005	23.4 ± 0.004	17.42 ± 0.02	20.01 ± 0.003	19.98 ± 0.02
α-Terpinolene	8.505	1.12 ± 0.01	1.57 ± 0.006	1.49 ± 0.02	1.24 ± 0.1	1.75 ± 0.001	1.62 ± 0.002	1.29 ± 0.004	1.87 ± 0.003	1.96 ± 0.002	1.16 ± 0.05	1.36 ± 0.005	1.33 ± 0.03
Linalool	8.769	0.60 ± 0.03	0.79 ± 0.004	0.71 ± 0.02	0.61 ± 0.0	0.87 ± 0.003	0.84 ± 0.03	0.61 ± 0.006	0.90 ± 0.006	0.97 ± 0.003	0.60 ± 0.003	0.68 ± 0.01	0.65 ± 0.005
Fenchol	8.963	0.04 ± 0.01	0.82 ± 0.001	0.64 ± 0.02	0.35 ± 0.03	0.95 ± 0.01	0.87 ± 0.002	0.48 ± 0.006	1.09 ± 0.06	1.19 ± 0.001	0.28 ± 0.002	0.55 ± 0.02	0.52 ± 0.004
Borneol	9.524	0.05 ± 0.002	0.26 ± 0.002	0.18 ± 0.03	0.07 ± 0.1	0.33 ± 0.03	0.31 ± 0.003	0.08 ± 0.002	0.41 ± 0.04	0.48 ± 0.02	0.06 ± 0.01	0.15 ± 0.05	0.12 ± 0.02
4-Terpineol	9.673	0.40 ± 0.003	0.83 ± 0.006	0.75 ± 0.003	0.51 ± 0.002	0.94 ± 0.003	0.88 ± 0.01	0.63 ± 0.001	1.00 ± 0.05	1.05 ± 0.006	0.46 ± 0.00	0.70 ± 0.003	0.67 ± 0.006
α-Terpineol	9.862	1.48 ± 0.01	1.69 ± 0.003	1.62 ± 0.006	1.51 ± 0.01	1.77 ± 0.005	1.74 ± 0.02	1.53 ± 0.001	1.82 ± 0.003	1.98 ± 0.007	1.50 ± 0.003	1.60 ± 0.006	1.57 ± 0.02
Nerol	10.096	0.85 ± 0.01	1.03 ± 0.02	0.97 ± 0.006	0.87 ± 0.01	1.11 ± 0.008	1.08 ± 0.04	0.87 ± 0.004	1.14 ± 0.005	1.27 ± 0.002	0.86 ± 0.006	0.94 ± 0.005	0.91 ± 0.01
Geraniol	10.525	0.42 ± 0.05	0.75 ± 0.03	0.67 ± 0.002	0.51 ± 0.02	0.86 ± 0.003	0.80 ± 0.003	0.57 ± 0.005	0.97 ± 0.03	0.99 ± 0.01	0.47 ± 0.004	0.64 ± 0.006	0.61 ± 0.03
α-Bisabolol	11.029	0.01 ± 0.04	0.17 ± 0.01	0.09 ± 0.002	0.03 ± 0.03	0.25 ± 0.01	0.22 ± 0.05	0.04 ± 0.032	0.28 ± 0.02	0.31 ± 0.003	0.02 ± 0.002	0.07 ± 0.001	0.04 ± 0.05
β-Santalol	11.298	0.08 ± 0.006	0.19 ± 0.003	0.16 ± 0.003	0.08 ± 0.04	0.32 ± 0.009	0.24 ± 0.002	0.05 ± 0.002	0.42 ± 0.01	0.58 ± 0.005	0.08 ± 0.001	0.12 ± 0.003	0.09 ± 0.003
Ledol	11.55	0.08 ± 0.005	0.23 ± 0.005	0.15 ± 0.004	0.08 ± 0.003	0.35 ± 0.01	0.28 ± 0.03	0.05 ± 0.02	0.58 ± 0.001	0.62 ± 0.003	0.08 ± 0.01	0.12 ± 0.04	0.09 ± 0.006
α-Bisabolol	11.773	0.18 ± 0.005	1.59 ± 0.006	1.50 ± 0.004	1.27 ± 0.02	1.67 ± 0.03	1.64 ± 0.004	1.37 ± 0.011	1.81 ± 0.003	1.98 ± 0.004	1.22 ± 0.003	1.44 ± 0.01	1.41 ± 0.001
β-Citronellal	11.99	0.05 ± 0.01	0.51 ± 0.004	0.42 ± 0.005	0.22 ± 0.02	0.69 ± 0.00	0.56 ± 0.03	0.31 ± 0.001	0.77 ± 0.002	0.85 ± 0.02	0.12 ± 0.04	0.38 ± 0.02	0.35 ± 0.002
α-Citral	12.185	1.98 ± 0.02	2.62 ± 0.005	2.51 ± 0.002	2.1 ± 0.003	2.70 ± 0.04	2.67 ± 0.02	2.4 ± 0.01	2.76 ± 0.01	2.95 ± 0.002	2.00 ± 0.01	2.47 ± 0.01	2.44 ± 0.01
β-Citral	12.305	1.76 ± 0.003	2.45 ± 0.004	2.37 ± 0.003	2.13 ± 0.01	2.57 ± 0.004	2.50 ± 0.04	2.23 ± 0.006	2.64 ± 0.006	2.76 ± 0.006	1.95 ± 0.01	2.30 ± 0.005	2.27 ± 0.04
Neryl acetate	13.215	2.20 ± 0.04	3.95 ± 0.02	3.72 ± 0.02	3.21 ± 0.003	4.11 ± 0.004	4.00 ± 0.001	3.57 ± 0.005	4.21 ± 0.003	4.30 ± 0.02	3.11 ± 0.003	3.64 ± 0.02	3.61 ± 0.01
*Trans*-geranyl acetate	13.907	0.40 ± 0.002	0.81 ± 0.16	0.70 ± 0.01	0.51 ± 0.02	0.99 ± 0.01	0.85 ± 0.02	0.58 ± 0.004	1.11 ± 0.005	1.25 ± 0.003	0.42 ± 0.001	0.65 ± 0.03	0.62 ± 0.003
δ-Elemene	17.466	0.23 ± 0.00	0.66 ± 0.01	0.58 ± 0.01	0.31 ± 0.01	0.74 ± 0.02	0.71 ± 0.05	0.43 ± 0.006	0.86 ± 0.003	0.88 ± 0.004	0.28 ± 0.02	0.50 ± 0.005	0.47 ± 0.02
α-Farnesene	17.741	0.41 ± 0.003	0.73 ± 0.01	0.66 ± 0.003	0.45 ± 0.003	0.81 ± 0.00	0.78 ± 0.003	0.55 ± 0.003	0.85 ± 0.01	0.93 ± 0.001	0.42 ± 0.01	0.62 ± 0.004	0.59 ± 0.003
β-Elemene	19.389	0.31 ± 0.004	0.55 ± 0.00	0.48 ± 0.003	0.37 ± 0.004	0.68 ± 0.1	0.60 ± 0.006	0.38 ± 0.02	0.76 ± 0.01	0.79 ± 0.004	0.34 ± 0.003	0.45 ± 0.02	0.42 ± 0.05
β-Caryophyllene	19.703	0.86 ± 0.01	1.65 ± 0.003	1.57 ± 0.004	1.12 ± 0.006	1.73 ± 0.003	1.70 ± 0.02	1.42 ± 0.01	1.86 ± 0.02	1.93 ± 0.01	0.95 ± 0.01	1.49 ± 0.003	1.46 ± 0.04
*Trans*-α-bergamotene	19.841	1.44 ± 0.19	2.69 ± 0.004	2.61 ± 0.004	2.32 ± 0.003	2.82 ± 0.01	2.74 ± 0.01	2.5 ± 0.002	3.00 ± 0.04	3.04 ± 0.003	2.00 ± 0.002	1.49 ± 0.01	2.54 ± 0.002
*Trans*-β-farnesene	21.162	0.16 ± 0.002	0.53 ± 0.005	0.42 ± 0.001	0.18 ± 0.003	0.69 ± 0.003	0.58 ± 0.01	0.29 ± 0.008	0.72 ± 0.005	0.88 ± 0.05	0.17 ± 0.003	0.36 ± 0.04	0.33 ± 0.003
α-Humulene	22.267	0.11 ± 0.03	0.53 ± 0.001	0.40 ± 0.001	0.19 ± 0.01	0.61 ± 0.01	0.58 ± 0.02	0.27 ± 0.009	0.67 ± 0.005	0.74 ± 0.02	0.14 ± 0.007	0.34 ± 0.01	0.31 ± 0.01
Germacrene-D	22.839	0.23 ± 0.02	0.45 ± 0.002	0.37 ± 0.00	0.25 ± 0.02	0.58 ± 0.01	0.50 ± 0.01	0.26 ± 0.005	0.61 ± 0.002	0.66 ± 0.005	0.24 ± 0.01	0.33 ± 0.005	0.3 ± 0.02
*Cis*-α-bisabolene	23.24	0.18 ± 0.003	0.46 ± 0.01	0.35 ± 0.02	0.20 ± 0.03	0.61 ± 0.01	0.51 ± 0.01	0.21 ± 0.002	0.68 ± 0.004	0.82 ± 0.003	0.18 ± 0.12	0.28 ± 0.01	0.25 ± 0.002
β-Selinene	24.035	0.02 ± 0.011	0.29 ± 0.02	0.21 ± 0.05	0.09 ± 0.04	0.37 ± 0.01	0.34 ± 0.002	0.12 ± 0.00	0.41 ± 0.009	0.49 ± 0.02	0.07 ± 0.003	0.19 ± 0.02	0.16 ± 0.003
β-Bisabolene	24.109	2.00 ± 0.002	3.82 ± 0.004	3.70 ± 0.04	3.13 ± 0.005	3.97 ± 0.00	3.87 ± 0.01	3.44 ± 0.001	4.10 ± 0.001	4.17 ± 0.01	2.95 ± 0.004	3.51 ± 0.03	3.48 ± 0.02
*Cis*-γ-bisabolene	24.201	0.06 ± 0.03	0.22 ± 0.007	0.14 ± 0.03	0.04 ± 0.003	0.30 ± 0.002	0.27 ± 0.02	0.04 ± 0.001	0.38 ± 0.005	0.42 ± 0.02	0.04 ± 0.004	0.11 ± 0.002	0.08 ± 0.01
*Trans*-γ-bisabolene	24.842	0.02 ± 0.05	0.29 ± 0.003	0.21 ± 0.06	0.09 ± 0.01	0.40 ± 0.003	0.34 ± 0.00	0.11 ± 0.006	0.45 ± 0.03	0.52 ± 0.003	0.05 ± 0.01	0.18 ± 0.00	0.15 ± 0.04
*Trans*-α-bisabolene	24.973	0.07 ± 0.005	0.37 ± 0.002	0.29 ± 0.01	0.09 ± 0.01	0.51 ± 0.01	0.42 ± 0.01	0.18 ± 0.05	0.57 ± 0.02	0.64 ± 0.011	0.08 ± 0.02	0.25 ± 0.01	0.22 ± 0.02
Germacrene-B	26.038	0.10 ± 0.006	0.66 ± 0.003	0.55 ± 0.003	0.35 ± 0.00	0.78 ± 0.01	0.71 ± 0.01	0.42 ± 0.003	0.84 ± 0.03	0.89 ± 0.003	0.24 ± 0.03	0.49 ± 0.012	0.46 ± 0.03
*p*-Menth-2-en-1-ol	26.232	0.05 ± 0.005	0.18 ± 0.001	0.10 ± 0.03	0.01 ± 0.01	0.32 ± 0.005	0.23 ± 0.03	0.01 ± 0.02	0.39 ± 0.00	0.55 ± 0.001	0.05 ± 0.01	0.08 ± 0.001	0.05 ± 0.03
Camphene hydrate	26.953	0.01 ± 0.13	0.49 ± 0.00	0.41 ± 0.06	0.07 ± 0.01	0.58 ± 0.002	0.54 ± 0.002	0.28 ± 0.01	0.62 ± 0.01	0.67 ± 0.00	0.04 ± 0.00	0.35 ± 0.02	0.32 ± 0.003
Isopulegone	31.227	0.02 ± 0.02	0.25 ± 0.003	0.17 ± 0.02	0.04 ± 0.02	0.37 ± 0.01	0.30 ± 0.003	0.04 ± 0.01	0.43 ± 0.01	0.49 ± 0.003	0.03 ± 0.004	0.11 ± 0.03	0.08 ± 0.005
Decanal	34.46	0.17 ± 0.03	0.46 ± 0.001	0.38 ± 0.01	0.14 ± 0.02	0.55 ± 0.02	0.51 ± 0.04	0.24 ± 0.004	0.62 ± 0.03	0.72 ± 0.001	0.18 ± 0.002	0.31 ± 0.005	0.28 ± 0.006
Bornyl acetate	34.689	0.02 ± 0.02	0.17 ± 0.002	0.13 ± 0.003	0.03 ± 0.2	0.30 ± 0.003	0.22 ± 0.01	0.03 ± 0.03	0.33 ± 0.02	0.38 ± 0.005	0.03 ± 0.001	0.10 ± 0.004	0.07 ± 0.004
Undecanal	34.827	0.03 ± 0.02	0.11 ± 0.001	0.09 ± 0.004	0.04 ± 0.003	0.15 ± 0.002	0.12 ± 0.01	0.05 ± 0.005	0.17 ± 0.01	0.19 ± 0.006	0.04 ± 0.001	0.07 ± 0.001	0.04 ± 0.001
Tetradecanal	34.891	0.12 ± 0.001	0.37 ± 0.01	0.29 ± 0.04	0.15 ± 0.004	0.50 ± 0.003	0.42 ± 0.002	0.19 ± 0.003	0.55 ± 0.001	0.62 ± 0.002	0.12 ± 0.012	0.26 ± 0.01	0.23 ± 0.02
Dodecanal	37.921	0.01 ± 0.001	0.04 ± 0.01	0.04 ± 0.01	0.01 ± 0.03	0.13 ± 0.01	0.09 ± 0.01	0.01 ± 0.02	0.17 ± 0.005	0.29 ± 0.002	0.02 ± 0.01	0.03 ± 0.01	0.03 ± 0.03

*D0: irrigation at 100% Fc, D1: irrigation at 75% Fc, D2: irrigation at 40% Fc, M0 (0 μM melatonin), M1 (50 μM melatonin). M2 (100 μM melatonin) and M3 (150 μM melatonin). The data were sorted based on the Retention Time (RT) of components. Values are given as mean ± SD (n = 3).*

**TABLE 4:** Essential oil compound of Persian lime, different concentrations of exogenous melatonin, and various levels of drought stress.

Compounds	RT	D0M0	D1M0	D2M0	D0M1	D1M1	D2M1	D0M2	D1M2	D2M2	D0M3	D1M3	D2M3
Nonane	3.859	0.002 ± 0.003	0.010 ± 0.003	0.008 ± 0.002	0.004 ± 0.003	0.012 ± 0.002	0.018 ± 0.002	0.005 ± 0.003	0.021 ± 0.005	0.032 ± 0.004	0.002 ± 0.003	0.006 ± 0.002	0.006 ± 0.002
Tricyclene	5.032	0.005 ± 0.02	0.006 ± 0.01	0.006 ± 0.01	0.004 ± 0.01	0.008 ± 0.003	0.007 ± 0.001	0.004 ± 0.001	0.009 ± 0.003	0.009 ± 0.002	0.005 ± 0.04	0.005 ± 0.01	0.005 ± 0.03
α-Thujene	5.633	0.561 ± 0.003	0.794 ± 0.02	0.734 ± 0.03	0.611 ± 0.02	0.847 ± 0.01	0.821 ± 0.02	0.662 ± 0.02	0.851 ± 0.01	0.875 ± 0.004	0.594 ± 0.05	0.712 ± 0.002	0.686 ± 0.002
α-Pinene	5.696	2.092 ± 0.003	3.532 ± 0.005	3.214 ± 0.004	2.243 ± 0.003	3.976 ± 0.02	3.742 ± 0.03	2.312 ± 0.04	4.032 ± 0.02	4.125 ± 0.02	2.110 ± 0.06	2.843 ± 0.01	2.641 ± 0.04
β-Pinene	5.794	10.921 ± 0.02	13.541 ± 0.04	13.021 ± 0.03	11.952 ± 0.4	13.921 ± 0.01	13.752 ± 0.01	13.201 ± 0.00	14.012 ± 0.02	14.147 ± 0.03	11.543 ± 0.04	12.874 ± 0.04	12.741 ± 0.02
Camphene	5.799	0.058 ± 0.003	0.064 ± 0.003	0.063 ± 0.002	0.059 ± 0.001	0.075 ± 0.03	0.071 ± 0.003	0.059 ± 0.00	0.080 ± 0.02	0.086 ± 0.01	0.058 ± 0.002	0.061 ± 0.01	0.060 ± 0.02
Sabinene	5.868	11.282 ± 0.001	11.376 ± 0.01	11.354 ± 0.01	11.294 ± 0.03	11.421 ± 0.02	11.416 ± 0.01	11.307 ± 0.04	11.432 ± 0.04	11.478 ± 0.03	11.290 ± 0.00	11.331 ± 0.01	11.310 ± 0.02
β-Myrcene	5.982	1.498 ± 0.005	1.746 ± 0.02	1.720 ± 0.02	1.499 ± 0.003	1.845 ± 0.002	1.831 ± 0.002	1.463 ± 0.003	1.930 ± 0.003	1.947 ± 0.004	1.486 ± 0.006	1.532 ± 0.001	1.510 ± 0.003
Octanal	6.108	0.053 ± 0.01	0.068 ± 0.005	0.064 ± 0.005	0.055 ± 0.01	0.075 ± 0.003	0.071 ± 0.001	0.058 ± 0.004	0.079 ± 0.001	0.084 ± 0.002	0.055 ± 0.01	0.060 ± 0.006	0.059 ± 0.005
α-Phellandrene	6.2	0.014 ± 0.02	0.022 ± 0.003	0.020 ± 0.04	0.015 ± 0.002	0.028 ± 0.001	0.026 ± 0.002	0.016 ± 0.002	0.033 ± 0.004	0.036 ± 0.01	0.014 ± 0.003	0.019 ± 0.003	0.019 ± 0.006
δ-3-Carene	6.429	0.009 ± 0.006	0.025 ± 0.00	0.022 ± 0.00	0.010 ± 0.004	0.032 ± 0.04	0.029 ± 0.04	0.011 ± 0.01	0.037 ± 0.005	0.042 ± 0.006	0.010 ± 0.002	0.018 ± 0.02	0.014 ± 0.004
α-Terpinene	6.52	0.288 ± 0.005	0.362 ± 0.004	0.352 ± 0.003	0.294 ± 0.02	0.412 ± 0.02	0.387 ± 0.005	0.298 ± 0.003	0.422 ± 0.006	0.471 ± 0.007	0.290 ± 0.004	0.321 ± 0.002	0.314 ± 0.02
*p*-Cymene	6.583	0.197 ± 0.02	0.287 ± 0.05	0.275 ± 0.004	0.214 ± 0.03	0.312 ± 0.03	0.310 ± 0.003	0.223 ± 0.002	0.341 ± 0.007	0.352 ± 0.004	0.199 ± 0.008	0.245 ± 0.04	0.232 ± 0.003
Limonene	6.755	45.203 ± 0.04	46.821 ± 0.04	46.723 ± 0.02	45.963 ± 0.01	47.125 ± 0.00	46.910 ± 0.00	46.102 ± 0.02	49.321 ± 0.01	51.402 ± 0.04	45.712 ± 0.05	46.552 ± 0.01	46.432 ± 0.02
(Z)-^a-ocimene	6.938	0.047 ± 0.003	0.059 ± 0.004	0.054 ± 0.003	0.049 ± 0.001	0.067 ± 0.002	0.063 ± 0.001	0.050 ± 0.002	0.072 ± 0.003	0.077 ± 0.004	0.048 ± 0.003	0.054 ± 0.006	0.052 ± 0.005
(E)-^a-ocimene	7.361	0.095 ± 0.002	0.106 ± 0.01	0.104 ± 0.01	0.98 ± 0.003	0.108 ± 0.006	0.108 ± 0.02	0.99 ± 0.004	0.110 ± 0.002	0.112 ± 0.003	0.096 ± 0.001	0.102 ± 0.005	0.100 ± 0.005
γ-Terpinene	7.401	10.323 ± 0.04	11.995 ± 0.02	11.621 ± 0.02	10.63 ± 0.00	12.220 ± 0.00	12.209 ± 0.01	10.75 ± 0.02	12.341 ± 0.03	12.456 ± 0.04	10.432 ± 0.02	11.312 ± 0.01	11.300 ± 0.02
***Cis***-sabinene hydrate^a^	7.556	0.034 ± 0.003	0.054 ± 0.003	0.052 ± 0.004	0.037 ± 0.002	0.062 ± 0.003	0.060 ± 0.003	0.039 ± 0.003	0.063 ± 0.002	0.065 ± 0.005	0.035 ± 0.003	0.045 ± 0.003	0.043 ± 0.02
Terpinolene	8.225	0.589 ± 0.004	0.682 ± 0.004	0.653 ± 0.004	0.597 ± 0.01	0.696 ± 0.01	0.692 ± 0.01	0.621 ± 0.01	0.711 ± 0.003	0.721 ± 0.004	0.593 ± 0.01	0.632 ± 0.01	0.628 ± 0.003
*Trans*-sabinene hydrate	8.357	0.041 ± 0.007	0.041 ± 0.005	0.041 ± 0.02	0.041 ± 0.004	0.041 ± 0.002	0.041 ± 0.02	0.041 ± 0.02	0.044 ± 0.001	0.045 ± 0.02	0.041 ± 0.005	0.041 ± 0.002	0.041 ± 0.01
Linalool	8.517	0.179 ± 0.008	0.187 ± 0.01	0.186 ± 0.03	0.180 ± 0.003	0.190 ± 0.004	0.191 ± 0.003	0.181 ± 0.004	0.195 ± 0.001	0.197 ± 0.003	0.180 ± 0.003	0.184 ± 0.006	0.182 ± 0.02
Nonanal	8.78	0.010 ± 0.001	0.022 ± 0.00	0.021 ± 0.001	0.017 ± 0.005	0.028 ± 0.004	0.026 ± 0.04	0.018 ± 0.006	0.031 ± 0.01	0.033 ± 0.004	0.015 ± 0.04	0.021 ± 0.04	0.020 ± 0.006
*Cis*-*p*-menth-2-en-1-ol^a^	8.975	0.005 ± 0.02	0.006 ± 0.01	0.005 ± 0.00	0.004 ± 0.02	0.007 ± 0.01	0.007 ± 0.005	0.005 ± 0.005	0.009 ± 0.02	0.009 ± 0.003	0.005 ± 0.006	0.005 ± 0.03	0.005 ± 0.002
*Trans*-pinocarveol	9.518	0.004 ± 0.03	0.005 ± 0.002	0.006 ± 0.003	0.004 ± 0.01	0.006 ± 0.02	0.005 ± 0.03	0.005 ± 0.001	0.007 ± 0.05	0.008 ± 0.005	0.004 ± 0.02	0.005 ± 0.002	0.005 ± 0.03
Citronellal	9.724	0.032 ± 0.01	0.035 ± 0.03	0.035 ± 0.01	0.034 ± 0.003	0.036 ± 0.05	0.035 ± 0.01	0.034 ± 0.001	0.038 ± 0.008	0.040 ± 0.005	0.033 ± 0.003	0.035 ± 0.006	0.036 ± 0.004
Borneol	9.885	0.020 ± 0.02	0.027 ± 0.04	0.027 ± 0.003	0.021 ± 0.05	0.031 ± 0.003	0.029 ± 0.006	0.021 ± 0.00	0.034 ± 0.007	0.036 ± 0.004	0.021 ± 0.01	0.024 ± 0.03	0.023 ± 0.006
Terpinen-4-ol	10.159	0.079 ± 0.01	0.084 ± 0.003	0.082 ± 0.01	0.081 ± 0.005	0.086 ± 0.002	0.085 ± 0.005	0.080 ± 0.003	0.093 ± 0.006	0.098 ± 0.001	0.079 ± 0.004	0.081 ± 0.004	0.081 ± 0.02
α-Terpineol	10.257	0.258 ± 0.003	0.471 ± 0.01	0.432 ± 0.02	0.372 ± 0.002	0.495 ± 0.01	0.496 ± 0.03	0.388 ± 0.01	0.503 ± 0.003	0.532 ± 0.001	0.323 ± 0.03	0.410 ± 0.002	0.401 ± 0.02
Dodecane^a^	10.394	0.016 ± 0.004	0.026 ± 0.02	0.024 ± 0.004	0.017 ± 0.003	0.031 ± 0.001	0.029 ± 0.004	0.019 ± 0.02	0.032 ± 0.001	0.034 ± 0.00	0.017 ± 0.04	0.022 ± 0.03	0.021 ± 0.004
Decanal	10.537	0.077 ± 0.003	0.082 ± 0.003	0.081 ± 0.002	0.078 ± 0.01	0.089 ± 0.005	0.086 ± 0.002	0.078 ± 0.01	0.093 ± 0.001	0.096 ± 0.006	0.078 ± 0.006	0.080 ± 0.04	0.079 ± 0.003
Nerol	11.04	0.104 ± 0.003	0.263 ± 0.04	0.259 ± 0.01	0.185 ± 0.02	0.290 ± 0.03	0.287 ± 0.001	0.216 ± 0.01	0.298 ± 0.00	0.302 ± 0.007	0.123 ± 0.01	0.253 ± 0.03	0.221 ± 0.05
Neral	11.155	1.119 ± 0.004	1.363 ± 0.02	1.358 ± 0.03	1.243 ± 0.001	1.385 ± 0.003	1.379 ± 0.003	1.264 ± 0.01	1.402 ± 0.02	1.412 ± 0.002	1.223 ± 0.003	1.321 ± 0.002	1.286 ± 0.04
Geraniol	11.315	0.048 ± 0.00	0.056 ± 0.003	0.053 ± 0.004	0.049 ± 0.003	0.060 ± 0.004	0.059 ± 0.01	0.050 ± 0.003	0.062 ± 0.04	0.067 ± 0.002	0.049 ± 0.04	0.054 ± 0.005	0.052 ± 0.01
Geranial	11.567	1.845 ± 0.01	1.879 ± 0.003	1.863 ± 0.05	1.848 ± 0.004	1.923 ± 0.005	1.912 ± 0.02	1.849 ± 0.002	1.951 ± 0.005	1.973 ± 0.00	1.846 ± 0.005	1.852 ± 0.01	1.851 ± 0.003
Bornyl acetate	11.784	0.003 ± 0.04	0.003 ± 0.02	0.003 ± 0.01	0.003 ± 0.02	0.003 ± 0.01	0.003 ± 0.006	0.003 ± 0.04	0.004 ± 0.005	0.007 ± 0.01	0.003 ± 0.004	0.003 ± 0.03	0.003 ± 0.001
Tridecane^a^	12.196	0.003 ± 0.003	0.003 ± 0.01	0.003 ± 0.00	0.003 ± 0.01	0.003 ± 0.001	0.003 ± 0.004	0.003 ± 0.02	0.005 ± 0.02	0.006 ± 0.02	0.003 ± 0.003	0.003 ± 0.02	0.003 ± 0.005
Undecanal	12.316	0.013 ± 0.006	0.022 ± 0.04	0.021 ± 0.003	0.017 ± 0.002	0.028 ± 0.002	0.026 ± 0.007	0.018 ± 0.006	0.030 ± 0.01	0.032 ± 0.06	0.014 ± 0.02	0.020 ± 0.004	0.0200.07
δ-Elemene	12.625	0.081 ± 0.007	0.089 ± 0.002	0.089 ± 0.005	0.082 ± 0.01	0.093 ± 0.02	0.091 ± 0.006	0.085 ± 0.006	0.095 ± 0.006	0.099 ± 0.003	0.085 ± 0.01	0.088 ± 0.03	0.087 ± 0.008
Neryl acetate	12.963	0.805 ± 0.01	0.945 ± 0.003	0.931 ± 0.01	0.841 ± 0.00	0.968 ± 0.03	0.963 ± 0.008	0.851 ± 0.03	0.972 ± 0.003	0.975 ± 0.008	0.823 ± 0.06	0.885 ± 0.02	0.862 ± 0.01
Geranyl acetate	13.14	0.175 ± 0.01	0.182 ± 0.005	0.181 ± 0.04	0.176 ± 0.003	0.185 ± 0.004	0.183 ± 0.002	0.178 ± 0.003	0.187 ± 0.02	0.189 ± 0.002	0.175 ± 0.01	0.179 ± 0.005	0.178 ± 0.02
â-Elemene	13.221	0.061 ± 0.02	0.072 ± 0.006	0.070 ± 0.05	0.064 ± 0.004	0.078 ± 0.01	0.077 ± 0.005	0.066 ± 0.004	0.080 ± 0.04	0.082 ± 0.003	0.062 ± 0.07	0.068 ± 0.03	0.069 ± 0.003
Dodecanal	13.913	0.044 ± 0.04	0.044 ± 0.002	0.044 ± 0.002	0.044 ± 0.02	0.044 ± 0.02	0.044 ± 0.003	0.044 ± 0.001	0.045 ± 0.05	0.046 ± 0.004	0.044 ± 0.008	0.044 ± 0.01	0.044 ± 0.01
*Trans*-R-bergamotene	14.737	1.026 ± 0.001	1.029 ± 0.03	1.027 ± 0.03	1.026 ± 0.01	1.033 ± 0.00	1.030 ± 0.005	1.026 ± 0.003	1.038 ± 0.006	1.045 ± 0.002	1.026 ± 0.003	1.026 ± 0.02	1.026 ± 0.02
(Z)-^a-farnesene	14.874	0.011 ± 0.003	0.024 ± 0.004	0.022 ± 0.001	0.015 ± 0.003	0.028 ± 0.003	0.026 ± 0.002	0.016 ± 0.005	0.030 ± 0.008	0.034 ± 0.003	0.013 ± 0.001	0.021 ± 0.003	0.018 ± 0.003
R-humulene	15.332	0.046 ± 0.002	0.052 ± 0.005	0.051 ± 0.005	0.045 ± 0.005	0.056 ± 0.05	0.054 ± 0.001	0.047 ± 0.02	0.059 ± 0.003	0.062 ± 0.003	0.046 ± 0.002	0.049 ± 0.004	0.048 ± 0.01
(E)-^a-farnesene	17.747	0.097 ± 0.005	0.097 ± 0.003	0.097 ± 0.03	0.097 ± 0.008	0.097 ± 0.006	0.097 ± 0.002	0.097 ± 0.04	0.098 ± 0.005	0.099 ± 0.01	0.097 ± 0.03	0.097 ± 0.005	0.097 ± 0.02
â-Santalene	19.395	0.037 ± 0.004	0.040 ± 0.003	0.041 ± 0.01	0.038 ± 0.008	0.043 ± 0.001	0.042 ± 0.06	0.038 ± 0.006	0.046 ± 0.007	0.049 ± 0.005	0.038 ± 0.02	0.039 ± 0.04	0.039 ± 0.003
Germacrene D	19.44	0.086 ± 0.01	0.091 ± 0.004	0.089 ± 0.04	0.087 ± 0.01	0.093 ± 0.002	0.091 ± 0.2	0.087 ± 0.03	0.097 ± 0.004	0.099 ± 0.006	0.086 ± 0.03	0.088 ± 0.05	0.088 ± 0.02
R-selinene	19.498	0.032 ± 0.02	0.041 ± 0.005	0.040 ± 0.003	0.035 ± 0.001	0.046 ± 0.03	0.043 ± 0.003	0.036 ± 0.05	0.049 ± 0.003	0.052 ± 0.001	0.033 ± 0.002	0.039 ± 0.003	0.037 ± 0.01
(Z)-R-bisabolene	19.635	0.123 ± 0.001	0.286 ± 0.01	0.275 ± 0.02	0.233 ± 0.01	0.311 ± 0.02	0.309 ± 0.001	0.243 ± 0.003	0.321 ± 0.003	0.331 ± 0.003	0.135 ± 0.03	0.264 ± 0.03	0.261 ± 0.003
(E,E)-R-farnesene +^a-bisabolene	19.841	1.710 ± 0.03	1.852 ± 0.02	1.849 ± 0.001	1.756 ± 0.02	1.876 ± 0.003	1.863 ± 0.04	1.810 ± 0.008	1.901 ± 0.01	1.920 ± 0.04	1.723 ± 0.02	1.843 ± 0.008	1.833 ± 0.003
Germacrene B	21.163	0.125 ± 0.002	0.212 ± 0.01	0.202 ± 0.01	0.143 ± 0.004	0.236 ± 0.005	0.226 ± 0.03	0.163 ± 0.008	0.241 ± 0.02	0.253 ± 0.03	0.137 ± 0.03	0.186 ± 0.03	0.174 ± 0.01
Tetradecanal	22.055	0.021 ± 0.003	0.026 ± 0.00	0.022 ± 0.00	0.020 ± 0.003	0.030 ± 0.004	0.028 ± 0.006	0.019 ± 0.001	0.035 ± 0.003	0.038 ± 0.04	0.021 ± 0.02	0.021 ± 0.03	0.021 ± 0.002
2,3-Dimethyl-3-(4-methyl-3-pentenyl)-2-norbornanol	22.278	0.046 ± 0.01	0.048 ± 0.003	0.048 ± 0.003	0.046 ± 0.01	0.050 ± 0.008	0.049 ± 0.003	0.046 ± 0.007	0.056 ± 0.003	0.059 ± 0.005	0.046 ± 0.004	0.046 ± 0.04	0.047 ± 0.01
Campherenol	22.633	0.054 ± 0.02	0.059 ± 0.004	0.053 ± 0.04	0.054 ± 0.02	0.063 ± 0.001	0.060 ± 0.002	0.054 ± 0.02	0.065 ± 0.002	0.067 ± 0.006	0.054 ± 0.03	0.055 ± 0.02	0.055 ± 0.02
R-bisabolol	22.662	0.067 ± 0.002	0.071 ± 0.02	0.069 ± 0.005	0.067 ± 0.005	0.072 ± 0.002	0.072 ± 0.004	0.068 ± 0.003	0.073 ± 0.005	0.074 ± 0.002	0.067 ± 0.01	0.068 ± 0.03	0.068 ± 0.004
Hexadecanal	22.73	0.078 ± 0.003	0.078 ± 0.03	0.078 ± 0.002	0.078 ± 0.01	0.079 ± 0.03	0.079 ± 0.01	0.079 ± 0.02	0.081 ± 0.009	0.083 ± 0.001	0.078 ± 0.002	0.079 ± 0.003	0.078 ± 0.002

*D0: irrigation at 100% Fc, D1: irrigation at 75% Fc, D2: irrigation at 40% Fc, M0 (0 μM melatonin), M1 (50 μM melatonin). M2 (100 μM melatonin) and M3 (150 μM melatonin). The data were sorted based on the Retention Time (RT) of components. Values are given as mean ± SD (n = 3).*

In Mexican lime nine major components including limonene (63.30 ± 0.005%), γ-terpinene (23.40 ± 0.004%), β-pinene (13.20 ± 0.01%), acetate neryl (4.30 ± 0.02%), β-bisabolene (4.17 ± 0.01%), α-pinene (3.72 ± 0.004%), *trans*-α-bergamotene (3.04 ± 0.003%), α-citral (2.95 ± 0.002%), β-myrcene (2.81 ± 0.006%), and β-citral (2.76 ± 0.006%) were detected. The minor components (<1%) including nerol (0.85 ± 0.01%), linalool (0.60 ± 0.03%), geraniol (0.42 ± 0.05%), 4-terpineol (0.40 ± 0.003%), α-terpinene (0.37 ± 0.00%), and δ-elemene (0.23 ± 0.00%) were also recognized in Mexican lime. Mexican lime has some exclusive terpenes such as the sesquiterpene, β-santalol (0.58 ± 0.005%) (Table 3). Generally, 21 sesquiterpenes, 13 monoterpenes, 12 terpene alcohols, 8 oxygen-containing aliphatics, 3 terpene aldehydes, 3 terpene esters, and 1 terpene ketone were identified in Mexican lime.

**FIGURE 5:**
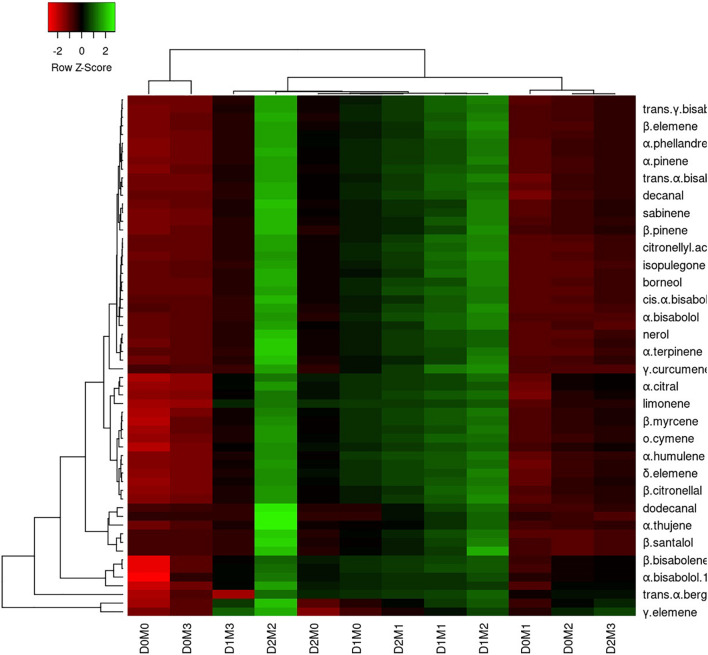
Heatmap representation of the interactive effects of drought stress and melatonin application on essential oil compound of Mexican lime. D0: irrigation at 100% FC, D1: irrigation at 75% FC, D2: irrigation at 40% FC, M0 (0 μM melatonin), M1 (50 μM melatonin). M2 (100 μM melatonin) and M3 (150 μM melatonin). Red and Green represent increased and decreased values, respectively. Values are given as mean ± SD (*n* = 3).

**FIGURE 6:**
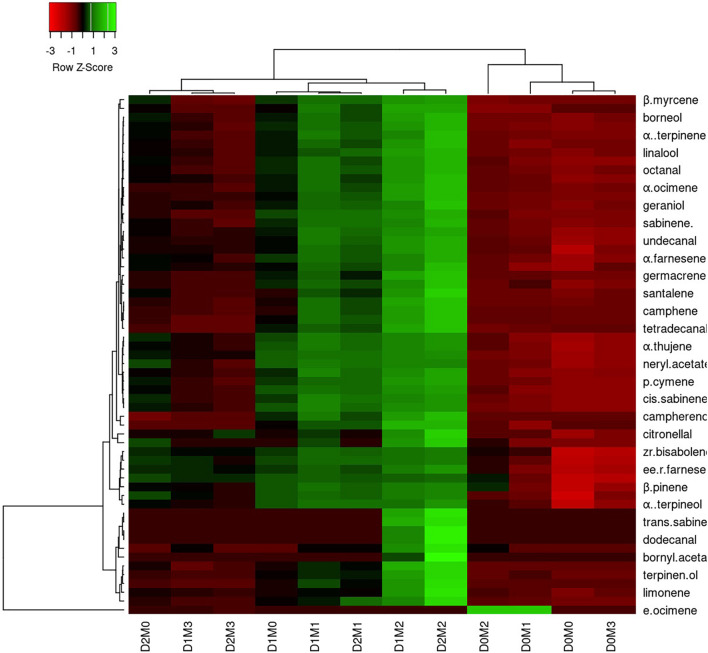
Heatmap representation of the interactive effects of drought stress and melatonin application on essential oil compound of Persian lime. D0: irrigation at 100% FC, D1: irrigation at 75% FC, D2: irrigation at 40% FC, M0 (0 μM melatonin), M1 (50 μM melatonin). M2 (100 μM melatonin) and M3 (150 μM melatonin). Red and Green represent increased and decreased values, respectively. Values are given as mean ± SD (*n* = 3).

In Persian lime 10 major detected components including limonene (51.40 ± 0.04%), β-pinene (14.14 ± 0.03%), γ-terpinene (12.45 ± 0.04%), sabinene (11.47 ± 0.03%), α-pinene (4.12 ± 0.02%), geranial (1.97 ± 0.00%), β-myrcene (1.94 ± 0.004%) (E,E)-R-farnesene +^a-bisabolene (1.92 ± 0.04%), neral (1.41 ± 0.002%), and *trans*-R-bergamotene (1.04 ± 0.002%) were identified (Table 4). According to the heat map (Figures 5, 6), the highest percentage of major essential oil components were obtained when both cultivars were exposed to 100 μM melatonin under severe drought stress (40% FC), in comparison with treatments that were under moderate drought stress (75% FC).

The various concentrations of exogenous melatonin remarkably influenced the essential oil components of both cultivars in different ways. Treating the two cultivars with 100 μM melatonin significantly led to limonene accumulation. Components such as α-terpinene and α-thujene showed a small increase in Mexican lime, while other components such as dodecanal and 1-terpinenol were constant. Also, components such as camphene and tricyclene showed a small rise in Persian lime, while other components such as hexadecanal and (E)-â-farnesene were constant. Essential oils were more pronounced under drought with 100 M melatonin addition, as seen in the heat map in Figures 5, 6.

## Discussion

In the current study, melatonin’s effect on the profile of secondary metabolites, including essential oil, total phenolics, polyphenols, and total flavonoids under drought stress, was investigated. Environmental conditions have a major impact on the biosynthesis of secondary metabolites including essential oils and phenolic compounds, in citrus plants. Drought stress reduces plant water absorption and water potentials, affecting a variety of physiological processes and altering secondary metabolite biosynthesis. Plants produce more secondary metabolites in drought conditions, which can be commercially advantageous to growers in arid and semi-arid regions (Valifard et al., 2014; Ashrafi et al., 2018).

Polyphenols, as one of the most important classes of secondary metabolites, play a significant role in different physiological processes (Winkel-Shirley, 2002; Treutter, 2006; Agati et al., 2012). Flavonoids and phenols can be considered another class of important secondary metabolites in plants, which have crucial functions in coping with various stressful environmental conditions (Di Ferdinando et al., 2014). Polyphenols, in general, are components of plants’ non-enzymatic antioxidant mechanism, which is activated in response to stress (Sarker and Oba, 2020). Based on the results of the current study, it can be concluded that leaf flavonoid compositions were significantly changed under drought stress. The greater accumulation of flavonoids contents in citrus under drought stress can be explained by higher activity of phenylalanine ammonia lyase (PAL) and higher levels of phenylpropanoids which catalyze the cinnamic acid production as a precursor of the flavonoids (Cabane et al., 2012). Moreover, flavonoid contents are remarkably influenced by plant growth regulators (Kim et al., 2009). For example, Singleton et al. (1999) reported that salicylic acid as a plant growth regulator resulted in increased flavonoid levels in common dandelion (*Taraxacum officinale*). Also, the results of polyphenolic compounds analysis in the present experiment showed that the total amount of phenolic compounds in the two *Citrus* cultivars was significantly increased by the application of melatonin under drought stress in comparison with the control treatment (plants without melatonin). In line with these results, Ezzo et al. (2018) and El-Awadi et al. (2017) reported that phenolic content accumulation was enhanced under abiotic stress. Improvement in the biosynthesis of the phenolic compounds might be due to the impact of drought conditions on different physiological and metabolic systems (Keutgen and Pawelzik, 2009). Furthermore, the promotive and positive effect of exogenous application of melatonin can be related to its signaling function through inducing different metabolic and physiological pathways and stimulating biosynthesis of different substances, preferably regulating under biotic/abiotic stresses (Tan et al., 2012). For instance, total phenolic content was significantly increased under drought stress in two genotypes of basil, including sweet basil (*Ocimum basilicum*) and basil (*Ocimum ciliatum*) (Ghasemi Pirbalouti et al., 2017), and avishan-e-denaee (*T. daenensis* Celak.) (Emami Bistgani et al., 2017b). Several studies illustrated that a significant increase in secondary metabolites contents such as flavonoids in Pea (*Pisum sativum*) (Nogués et al., 1998), and total phenols in milfurada (*Hypericum brasiliense*) (Abreu et al., 2008), kacip Fatimah (*Labisia pumila* Benth. & Hook.) (Jaafar et al., 2012), peach (*Prunus persica* L.) (Kubota, 1988), ajowan caraway (*Trachyspermum ammi* L.) (Azhar et al., 2011), and purple Cone Flower (*Echinacea purpurea* L.) (Gray et al., 2003) was achieved under drought stress. The results of the current study showed that exposing two lime cultivars to melatonin under drought stress resulted in increasing total phenolic and flavonoids contents (Table 1) in compared with the control treatment. Similar results have been previously reported by Liang et al. (2018) on kiwifruit (*Actinidia chinensis*). Their results showed that amount of flavonoids accumulated in seedlings pretreated with melatonin, and transcript levels of eight genes involved in flavonoid synthesis, including PAL, were enhanced in response to melatonin application. These results indicated that melatonin delayed aging of kiwifruit leaves by activating the antioxidant capacity and enhancing flavonoid biosynthesis. In another studies, Bahcesular et al. (2020) on basil (*O. basilicum* L.), and Naghizadeh et al. (2019) on moldavian balm (*D. moldavica* L.) observed that foliar application of 100 μM melatonin increased secondary metabolites synthesis in plant under moderate and severe drought stress probably through regulation of secondary metabolism and the enzymes activity of PAL and polyphenol oxidase. The exogenous application of melatonin can improve plant antioxidant ability by enhancing antioxidant enzyme activities and alleviating leaf senescence by improving flavonoid production (Yin et al., 2013; Ben Abdallah et al., 2016; Liang et al., 2018). The HPLC analysis results of the current study illustrated the presence of phenolic components in both extracts were quantified and approved by analytical standard curves. The findings of the present study showed that hesperidin and eriocitrin were the major components in both extracts. In line with these results, Peterson et al. (2006) reported that hesperidin (15.64 mg/100 g DW) and eriocitrin (1.38 mg/100 g DW) were the main components of Mexican lime. Xu et al. (2010) showed that the level of proanthocyanidins, total phenols, anthocyanins, and flavonoids had a significant correlation with the antioxidant characteristics of plants. Also, Zhang and Tsao (2016) demonstrated that polyphenols play a significant role in the antioxidant properties of grape berries. Based on the best of authors’ knowledge, there are no studies to link the level of drought stress, melatonin contents directly, and polyphenol contents in citrus; therefore, the current study can provide the first document that melatonin increased the antioxidant ability through improving the accumulation of polyphenols. In another study (Peleg et al., 1991), results derived from HPLC chromatogram showed that gallic acid (212.4 ± 0.02 μg/g DW), pyrogallol (541.27 ± 0.03 μg/g DW), syringic acid (269.04 ± 0.05 μg/g DW), and caffeic acid (249.9 ± 0.05 μg/g DW) were detected as the main phenolic components in bitter orange (*Citrus aurantium*) bloom. In comparison, rutin (362.8 ± 0.02 μg/g DW) and naringin (688.1 ± 0.05 μg/g DW) were identified as the main flavonoid components. These results were reported by Peleg et al. (1991), who showed that caffeic acid and gallic acid, were the main phenolic compounds in *Citrus* species. The modulation of the phenylpropanoid biosynthetic process can be considered as the major reason for the drought-induced phenolic compound accumulation. Indeed, several key genes involved in the phenylpropanoid pathway are regulated by drought stress, which leads to stimulating the phenolic compound biosynthesis (Hernández et al., 2009). The phenylpropanoid pathway can be categorized as one of the most important secondary metabolic pathways that play a key role in plant defense mechanisms against abiotic stresses (Sharma et al., 2019), and the phenolic components that contribute to the plant’s resistance to drought stress consisting of phenolic acids, flavones, and flavonoids (Ballizany et al., 2012; Li et al., 2018; Rezayian et al., 2018; Gharibi et al., 2019).

Essential oils can be considered natural products whose pattern of composition, yield, and the level of individual compounds are related to several extrinsic and intrinsic factors. Moreover, the quality and quantity of these biomolecules are influenced by ecological and environmental conditions (Zarei et al., 2015). The results of the current study showed that there was a greater increase in the production of essential oils in both Mexican lime and Persian lime under drought stress in comparison with control treatment. Similar results were also reported by Dunford and Vazquez (2005). Moreover, in line with our results, Simon et al. (1992) revealed that an increase in the essential oil accumulation might be due to the higher density of essential oil secretory cavities under drought stresses, which ultimately resulted in the leaf area shrinkage. Alternatively, in the treatments under moderate drought stress, decreasing essential oil levels may relate to the storage of these components in the glandular trichomes in the leaf blade (Khalid, 2006). The absolute gland number produced prior to leaf emergence through the early epidermal cell divisions of leaves can also be increased under drought stress (Karray-Bouraoui et al., 2010). Turtola et al. (2003) demonstrated that a trade-off between defense and growth resulted in minor carbon allocation to growth which ultimately leads to stimulate the production of terpene as an essential oil under drought stress in rosemary leaves. Several studies showed that drought stress resulted in higher production of essential oils in different plants such as rosemary (*Rosmarinus officinalis* L.) (Abbaszadeh et al., 2020), six *Lamiaceae* species (García-Caparrós et al., 2019), basil (*O. ciliatum*) (Abdollahi Mandoulakani et al., 2017), avishan-e-denaee (*T. daenensis* Celak.) (Emami Bistgani et al., 2017a), and garden thyme (*Thymus vulgaris*), and *T. daenensis* (Alavi-Samani et al., 2015). Based on the results of the current study, the suitable level of exogenous melatonin significantly reduced the negative impact of drought stress through improving physiological and morphological responses and increasing the quality and quantity of essential oils. The function of melatonin in essential oil production in plants has not been well studied. However, the similarity between melatonin and indole-3-acetic acid (IAA) in chemical structure (both derived from chorismate) and bio-function (promoting essential oil biosynthesis) can be proposed as one of the possible mechanisms (Hazzoumi et al., 2014; Wang et al., 2016). Also, Silva et al. (2005) showed that an increase in essential oil production of *Salvia* species in response to exogenous application of melatonin might be due to the potential improvements of meristematic cells and site of biosynthesis of several chemical components that are crucial for essential oil productions. Based on the findings of the current study, it can be concluded that the foliar application of melatonin may regulate the feed-back of shikimic acid and tryptophan biosynthesis pathway, which are necessary for the biosynthesis of some metabolites such as limonene and methyl *N*-methylanthranilate (Munné-Bosch and Peñuelas, 2003; Xu et al., 2011). Among the detected essential oils in both lime cultivars (Tables 3, 4), the major essential oil of Mexican lime were limonene, terpinene, β-pinene, acetate neryl, β-bisabolene, γ-terpinene, α-pinene, *trans*-α-bergamotene, α-citral, β-myrcene, and β-citral, while the main essential oil compounds of Persian lime were limonene, β-pinene, γ-terpinene, sabinene, α-pinene, geranial, β-myrcene (E,E)-R-farnesene +â-bisabolene, neral, and *trans*-R-bergamotene.

Based on the results of the present study (Tables 3, 4), the four main essential oil compounds in all studied treatments were γ-terpinene, Limonene, α-pinene, and β-pinene. In line with these results, Alfonzo et al. (2017) and Sun et al. (2018) reported that although limonene was the major essential oil component of two lime cultivars, the limonene level considerably varied among different cultivars under different environmental conditions. Limonene can be categorized as a single-cyclic terpenoid with a bitter taste and strong citrus odor (Eldahshan and Halim, 2016). Also, Amorim et al. (2016) have previously reported that γ-terpinene, Limonene, α-pinene, and β-pinene were the main essential oils of Mexican lime grown in Rio de Janeiro State. Drought stress and foliar application of melatonin increased all secondary metabolites in this study. Therefore, the use compounds might also cause these results. The majority of the investigated compounds are formed by a single metabolic pathway and have identical precursors. When two compounds share the same precursor, improvements in growth conditions that improve the production of one of them can have a synergistic effect on the production of the other (Gharibi et al., 2016).

## Conclusion

In the current study, the foliar application of melatonin under drought stress was evaluated on total flavonoid, total phenolic, essential oil, and polyphenol compounds of two *Citrus* species. One of the most effective strategies for increasing plant tolerance to stress conditions is the foliar application of growth regulators. Stress promoted the synthesis of secondary metabolites, resulting in more essential oil and phenolic compound extraction from stressed plants compared to well-watered plants. As a result, plants grown under stress and melatonin treatment produced the highest essential oil content and phenolic compound. Overall, with regard to the results of this study, the extract of citrus leaves could be an important source of phenolic compounds and essential oils with antioxidant capacity. Finally, it could be concluded that foliar application of melatonin under drought stress, as a possible approach, can be used to increase the phenolic compounds and antioxidant activity in arid and semiarid areas.

## Data Availability Statement

The original contributions presented in the study are included in the article/supplementary material, further inquiries can be directed to the corresponding author/s.

## Author Contributions

MJ performed the experiments, analysis and interpretation of data, summed up, and wrote the manuscript. AS designed and lead the experiments, supervision, and revised the manuscript. Both authors contributed to the article and approved the submitted version.

## Conflict of Interest

The authors declare that the research was conducted in the absence of any commercial or financial relationships that could be construed as a potential conflict of interest.

## Publisher’s Note

All claims expressed in this article are solely those of the authors and do not necessarily represent those of their affiliated organizations, or those of the publisher, the editors and the reviewers. Any product that may be evaluated in this article, or claim that may be made by its manufacturer, is not guaranteed or endorsed by the publisher.
